# Inferring Clonal Composition from Multiple Sections of a Breast Cancer

**DOI:** 10.1371/journal.pcbi.1003703

**Published:** 2014-07-10

**Authors:** Habil Zare, Junfeng Wang, Alex Hu, Kris Weber, Josh Smith, Debbie Nickerson, ChaoZhong Song, Daniela Witten, C. Anthony Blau, William Stafford Noble

**Affiliations:** 1 Department of Genome Sciences, University of Washington, Seattle, Washington, United States of America; 2 Division of Hematology, Department of Medicine, University of Washington, Seattle, Washington, United States of America; 3 Department of Biostatistics, University of Washington, Seattle, Washington, United States of America; 4 Department of Computer Science and Engineering, University of Washington, Seattle, Washington, United States of America; Weizmann Institute of Science, Israel

## Abstract

Cancers arise from successive rounds of mutation and selection, generating clonal populations that vary in size, mutational content and drug responsiveness. Ascertaining the clonal composition of a tumor is therefore important both for prognosis and therapy. Mutation counts and frequencies resulting from next-generation sequencing (NGS) potentially reflect a tumor's clonal composition; however, deconvolving NGS data to infer a tumor's clonal structure presents a major challenge. We propose a generative model for NGS data derived from multiple subsections of a single tumor, and we describe an expectation-maximization procedure for estimating the clonal genotypes and relative frequencies using this model. We demonstrate, via simulation, the validity of the approach, and then use our algorithm to assess the clonal composition of a primary breast cancer and associated metastatic lymph node. After dividing the tumor into subsections, we perform exome sequencing for each subsection to assess mutational content, followed by deep sequencing to precisely count normal and variant alleles within each subsection. By quantifying the frequencies of 17 somatic variants, we demonstrate that our algorithm predicts clonal relationships that are both phylogenetically and spatially plausible. Applying this method to larger numbers of tumors should cast light on the clonal evolution of cancers in space and time.

## Author Summary

Cancers arise from a series of mutations that occur over time. As a result, as a tumor grows each cell inherits a distinctive *genotype*, defined by the set of all somatic mutations that distinguish the tumor cell from normal cells. Acertaining these genotype patterns, and identifying which ones are associated with the growth of the cancer and its ability to metastasize, can potentially give clinicians insights into how to treat the cancer. In this work, we describe a method for inferring the predominant genotypes within a single tumor. The method requires that a tumor be sectioned and that each section be subjected to a high-throughput sequencing procedure. The resulting mutations and their associated frequencies within each tumor section are then used as input to a probabilistic model that infers the underlying genotypes and their relative frequencies within the tumor. We use simulated data to demonstrate the validity of the approach, and then we apply our algorithm to data from a primary breast cancer and associated metastatic lymph node. We demonstrate that our algorithm predicts genotypes that are consistent with an evolutionary model and with the physical topology of the tumor itself. Applying this method to larger numbers of tumors should cast light on the evolution of cancers in space and time.

## Introduction

Many clones exist within each cancer, and selective pressure imposed by environmental factors, most notably treatments directed at tumor eradication, favors the emergence of clones that grow increasingly resistant to successive rounds of therapy. Incorporating this intra-tumor heterogeneity into strategies for planning, monitoring, and revising cancer treatment could improve outcomes for oncologists and their patients. Therefore, methods for estimating the number, size and mutational content of clones within a patient's tumor are being explored.

New approaches are being developed to assess the clonal content of a given tumor. Methods based on the interrogation of individual cells have relied on the use of fluorescent markers [1], [2] or single cell sequencing [3]–[6]. Whereas fluorescence-based approaches are inevitably limited by the relatively small number of features they can accommodate, single cell sequencing brings the highest possible resolution to characterizing an individual patient's tumor. Nonetheless, single cell sequencing also faces obstacles to its widespread implementation. Evaluating sufficiently large numbers of single cells to obtain statistical power can be prohibitive, for technical or financial reasons. Additionally, it is often difficult to ascertain the identity of the cells being sequenced, and details regarding the spatial positioning of cells relative to each other and to other cells in the tumor are lost when the single cells are obtained. These disadvantages pose significant challenges to the widespread adoption of single cell sequencing as a means for assessing tumor heterogeneity.

Complementing single cell approaches are efforts to deconvolve clonal subpopulations based on the frequencies of mutated alleles within one or more bulk tumor specimens. Shah et al. [7], who sequenced a breast cancer at the time of diagnosis and nine years later, after metastasis, pointed out that allele frequencies of the mutations shared between the two samples could be used to segregate primary mutations into those that occur in a dominant versus subdominant clone. This insight is the basis for a variety of approaches that apply clustering algorithms to mutation allele frequencies, including kernel density estimation [8] and Dirichlet process modeling applied either to the allele frequencies [9] or to a combination of allele frequency, loss-of-heterozygosity status and copy number [10]–[13].

Clearly, statistical power to infer variants and, ultimately, clonal composition, is increased if multiple samples are available for analysis. Accordingly, various studies have examined the progression of cancer within one or more patients over time. Sets of variants that exhibit similar allele frequencies within a single sample are suggestive of a clonal population. Hence, clustering methods to identify groups of mutations associated with a single clone have been applied. For example, kernel density estimation has been applied to allele frequencies from tumor-relapse pairs from eight acute myeloid leukemia (AML) patients [14] and from seven secondary AML patients [15].

An orthogonal approach taken by Newberger et al. [16] employs triplet samples of neoplasia, matched normal and carcinoma from six patients to infer lineages of various genetic events. They characterize each locus in terms of a binary vector representing the presence of the mutation across the various samples and then group the loci into classes on the basis of these vectors. After filtering low frequency classes, the classes are used to manually construct a phylogenetic tree. The focus of the study is to identify the shared characteristics of the evolutionary process across six patients with breast cancer.

**Figure 1:**
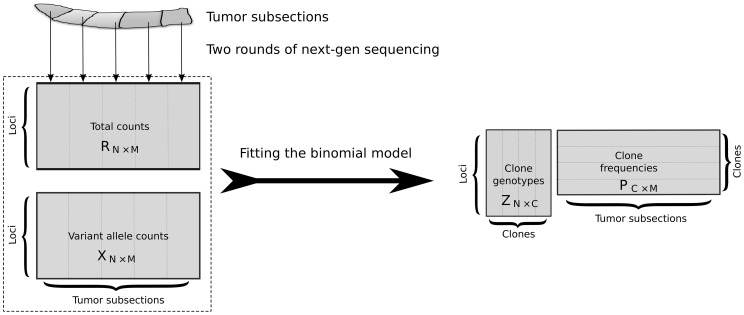
Inference of tumor clonal content. A collection of subsections of a tumor are subjected to next-generation sequencing to measure, across a common set of genomic loci, counts of two alleles—the *normal* allele that was observed in a matched normal sample at that locus, and a *variant allele*. The resulting counts matrices are provided as input to an inference procedure that estimates the clonal genotypes and frequencies.

In the current study, we adopt an alternative approach to identify clonal structure. Rather than measuring allele frequencies in multiple samples from the same patient over time, we physically subdivide a single breast cancer specimen and measure allele frequencies within each subsection (Figure 1). We are aware of two previous studies that have adopted such an approach. Yachida et al. [17] analyzed seven metastatic pancreatic cancers, sequencing from multiple samples per patient. Clones are initially defined relative to sample types (peritoneal, liver and lung metastases). Subsequently, the tumors from two patients are resected and a clonal phylogeny is inferred manually. More recently, Gerlinger et al. [18] carried out exome sequencing followed by targeted deep sequencing on samples from four patients with renal carcinoma. Each primary tumor was divided into 9 regions, and a phylogeny was manually constructed by assuming that higher alternate allele frequencies correspond to earlier mutations. In neither of these studies was an algorithm proposed to automatically infer from such data both the clonal genotypes and the relative frequencies of the clones within each subsection.

The method proposed here bears some similarity to the recently proposed Tree Approach to Clonality (TrAp) method [19]. The TrAp algorithm aims to identify the number, relative frequencies and genotypes of clones within a tumor using a formalism somewhat similar to ours, based on matrix decomposition. However, rather than analyzing data from multiple sections, the authors use as input a single set of variant allele frequencies and then constrain the resulting optimization problem by introducing a series of four assumptions about cancer evolution. It is not clear whether the method can easily generalize to analysis of data from multiple sections or multiple time points.

Here we describe a generative binomial model that incorporates information from multiple sections from a single tumor at a single time point to infer the frequencies and genotypes for a specified number of clones. An implementation of our algorithm is available through Bioconductor as an R package called Clomial (http://www.bioconductor.org/packages/release/bioc/html/Clomial.html). We use Clomial version 1.1.7 to apply this approach to a breast cancer specimen and demonstrate that the results from our model predict relationships that are phylogenetically and spatially plausible.

## Results

### 1 Inferring the clonal architecture of a tumor

We assume that a tumor is comprised of multiple populations of cells (“clones”), each with a unique genotype, and that these populations are heterogeneously distributed within the tumor itself. We collect, from several physical subsections of the tumor, shotgun sequencing reads. We also collect sequencing data from a non-tumor subsection from the same patient. Using the called genotypes from the normal subsection, and restricting ourselves to positions that are homozygous in the normal subsection, each read from a tumor subsection exhibits either a normal allele or a variant allele at each location. We exclude positions that exhibit homozygous normal alleles in all of the tumor subsections. Our goal is to infer, from the remaining 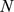 mutated positions, the genotype of each clonal population and their relative frequencies within each physical subsection of the tumor.

Formally, the problem can be stated as follows. Note that we use bold face letters for random variables, and that 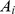 and 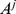 respectively denote the 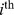 row and the 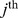 column of matrix 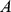. We are given two primary input matrices 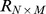 and 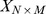, where 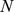 is the number of mutated loci, 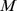 is the number of subsections (of which one is normal and 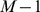 are tumor), 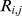 is the total number of reads (i.e., the coverage) at locus 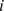 in subsection 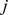, and 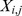 is the number of cancerous reads (those supporting the mutation) at locus 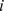 in subsection 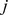. We assume, without loss of generality, that the first of the 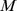 subsections corresponds to normal tissue, and that the remaining 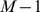 subsections are from the tumor. In addition, we consider 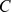, the number of distinct clones in the tumor, as a hyperparameter, and train a model based on a given value of 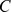. We assume that the first clone corresponds to the normal cell population and the tumor is composed of 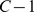 tumor clones. Later, we will discuss whether 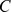 can be estimated from the data. Our task is to infer two matrices: a *clone frequency matrix* 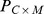 in which 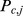 is the proportion of cells of clone 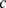 in subsection 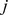, and a *genotype matrix* 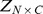 in which 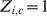 if clone 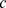 has the variant allele at locus 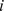, and 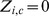 otherwise. The first column of 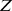 contains all zeroes because it represents the “normal clone.” By definition, each column of 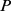 sums to 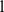. Also, by construction, the first column of 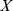 corresponds to the normal subsection and hence consists almost entirely of zeroes, although small non-zero counts may be possible due to contamination from tumor or due to sequencing error. If the first column of 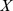 consisted entirely of zeroes, then we would expect the first column of 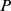 to be of the form 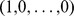, but in order to allow for the possibility that the allegedly normal subsection can have slight tumor contamination, we infer the first column of 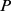 (as well as the other 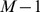 columns).

We propose to solve this problem using a generative model whose parameters are learned via expectation-maximization (EM) [20]. Accordingly, we define a matrix of hidden variables 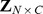 representing the unknown genotypes of the clones; for instance, if 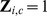, then the 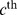 clone has a tumor allele at the 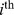 locus. We assume that each 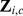 follows an independent Bernoulli distribution with parameter 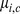, i.e.,
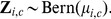
(1)


We also assume that if a mutation is present in a particular clone, then at that locus the clone is heterozygous with copy number equal to 1. Therefore, for subsection 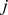, if clone 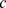 has a mutation at locus 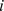 (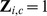), then its contribution to the observed count of cancer alleles is by 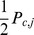, half of its proportion in the subsection. Conversely, if a clone does not have a mutation at 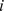 (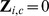), then it does not contribute to the count of variant alleles. By summing up the contributions of all clones, we obtain the total probability that an observed read corresponds to a variant allele rather than a normal allele. Therefore, the probability that a read contains the variant allele at locus 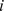 in subsection 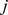 is given by
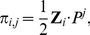
(2)where 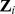 is the 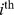 row of 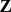, and 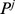 is the 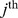 column of 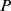. Finally, we introduce a matrix 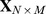 of random variables representing the observed data, where 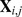 is the number of reads exhibiting the variant allele at locus 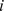 in subsection 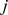. This matrix encodes our primary assumption about the distribution of the data: for each 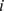 and 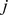, we observe an independent sample of 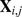 that has a binomial distribution with two parameters 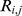 and 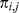, i.e.,
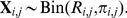
(3)


The first parameter of this distribution 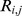 is the (known) total number of reads at locus 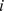 in subsection 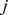. The second parameter, 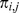, is the probability of observing a variant allele; it will be inferred by EM.

Given the joint distribution 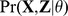 over observed variables 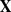 and latent variables 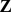, governed by parameters 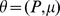, our goal is to maximize the likelihood function 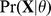. We do so using EM, exploiting three assumptions: (1) that each subsection contains non-zero normal contamination, i.e., 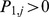 for all 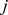, (2) independence of the 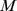 subsections from each other, and (3) independence of mutations from each other. The first assumption is based on the widely accepted difficulty associated with obtaining perfectly pure samples of tumor cells [21], [22]. The two independence assumptions essentially state that each locus and each sample is informative. These assumptions are unavoidable: in the presence of very high dependence, only very limited information about the underlying clonal composition of the tumor would be provided by the loci and samples. Furthermore, it is worth noting that these independence assumptions are made *conditional* on the parameters in the model: that is, the elements of 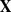 are independent conditional on 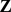 and 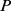. In other words, if we knew the true underlying parameters for the model (that is, the true genotypes for the clones, and the true proportion of each clone present in each sample), then the actual number of “tumor” reads that we would observe for each locus-sample pair would be independent.

While the formulation of our inference problem shows some similarity to well-studied matrix factorization problems [23]–[25], such techniques cannot be directly applied here. Unlike most matrix factorization techniques, which assume a normal distribution, our observations are binomially distributed. Moreover, the elements of the latent matrix 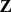 are binary, and each column of 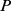 must sum to 1. These constraints required us to develop a customized inference algorithm.

### 2 Log likelihood

To frame the EM optimization, we consider the following complete-data log likelihood function of the model:
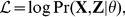
(4)which can be computed as follows (for details see Note S4 in Text S1):
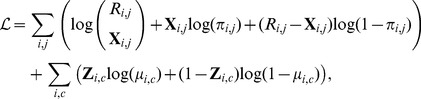
(5)where 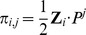.

### 3 Expectation maximization (EM) algorithm

Our goal is to find the parameters 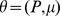 which maximize the likelihood. Because our model involves the hidden variable 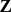, we cannot directly maximize the 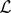 given in Equation 5 with respect to 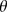. Instead, we use the EM algorithm to fit the model to the data [26]. EM is an iterative algorithm with two steps—E (for expectation) and M (for maximization)—in each iteration. In the E step, we use the current estimates of the parameters, 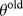, to compute the conditional expectation of 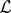. In the M step, we find the new parameters 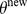 that maximize the conditional expectation.

#### Overview

In this section, we present an overview of the EM algorithm, followed by the specific details of the E and M steps for our application.

1. Randomly initialize the parameters 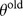.
2. Repeat the following until a convergence criterion is satisfied (such as insignificant improvement in the log likelihood; see Equation 12).
  - **E Step.** Evaluate the posterior 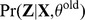 using the current parameter values. Because each locus is independent, we will compute 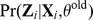 for 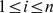. This can be done by Bayes' theorem,
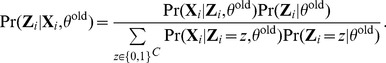
(6)
  - **M Step.** Evaluate 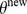 from
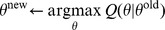
where 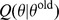 is the following expected log likelihood with respect to 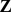 conditioned on 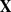 and 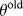:
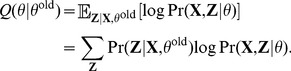
(7)
  - Update the parameters by
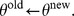

#### Computation for the E step

To compute the posterior 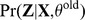, we need to compute 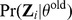 and 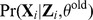 for the 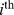 locus (see Equation 6). The latter is equal to the product of binomial probabilities because the samples are assumed to be independent. Using Equations 2 and 3, we have
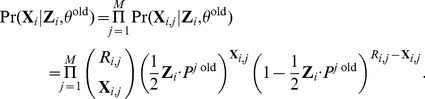
(8)Also, 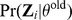 is the product of Bernoulli probabilities. From Equation 1, we have that
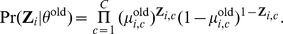



#### Computation for the M step

To get 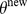, we maximize 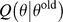 defined in Equation 7. First, we split 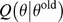 into two terms such that one term depends only on 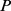, and the other term depends only on 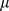. This simplifies the process of finding the optimal parameters.
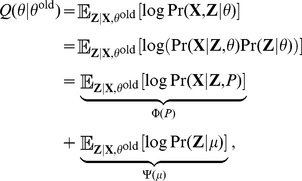
(9)where we let 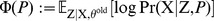 and 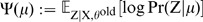 for simplicity of notation. Similar to Equation S7 in Note S4 in Text S1, we have used the fact that conditional on 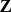 and 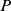, 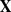 is independent of 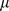, as well as the fact that conditional on 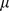, 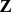 is independent of 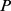.

**Computing** 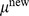 Now that we have separated 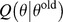 into two terms, we can first update 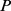 by only maximizing 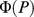. We solve the following constrained optimization problem to get 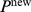 (for details see Note S5 in Text S1):
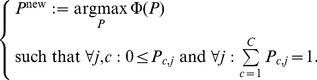
(10)


We solve our simplified optimization problem using a quasi-Newton method called BFGS-B [27], [28]. The original BFGS algorithm uses the gradient to approximate the Hessian matrix of second derivatives; therefore, the algorithm is very efficient when the gradient is available [29], [30]. BFGS-B is a variant that can handle simple box constraints. We compute the first derivative of 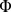 with respect to each entry of 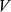 by the chain rule, and provide it to BFGS-B for faster convergence (Note S2 in Text S1).

**Computing** 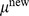 Recall that 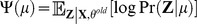 is the only part of the expected log likelihood which is a function of 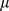 (see Equation 9). Therefore, we can compute 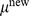 by maximizing 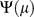. Because we are assuming that conditional on 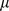, the elements of 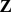 are independent, we just need to maximize each term in the following sum:
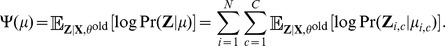



The first column of 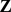 corresponds to the normal cells, 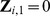, hence 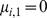 for all 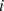. For 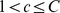, we need to maximize, with respect to 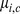,
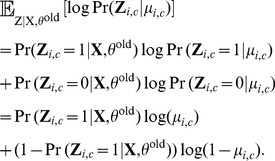
(11)


By single-variable calculus, the value of 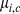 that maximizes (11) is 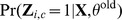. For the 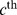 clone, the probability 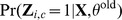 can be computed by marginalizing 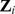 over all other clones:
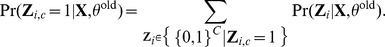



Because the posteriors 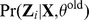 are easy to compute by Bayes' rule (Equation 6), 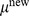 can be updated as follows:
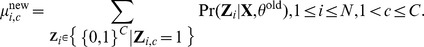



In principle, for each solution, the genotype matrix 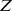 can be obtained by rounding the inferred 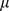. However, in practice, the inferred values in 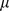 were always exactly 0 or 1 (with observed differences 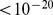).

#### Initialization and convergence

We initialize elements of 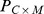 with values independently sampled from a Uniform 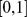 distribution. Then we standardize each column such that the sum of the proportions of each clone in a subsection is 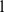. Similarly, we randomly initialize the matrix 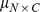 with values independently sampled from a Uniform 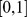 distribution. In practice, we run EM to convergence from multiple random initializations for 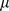 and 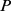, and we choose the run that results in the highest likelihood.

The convergence criterion is based on the change in the expectation of the complete-data log likelihood. Specifically, we stop the EM iterations if:
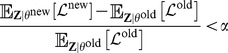
(12)where 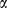 is a small positive number. We set 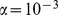 in our experiments. Using Equation 5, we can compute 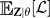 in each iteration as follows:
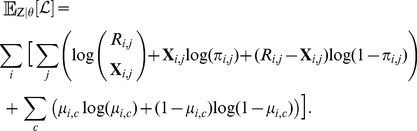
(13)where the sums are over locus indices 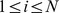, subsection indices 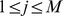, and clone indices 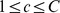. We used the fact that 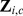 is binary and 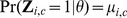 to derive the above equation.

### 4 Simulation results

To validate our implementation of the EM optimization procedure and to understand our model's behavior, we produced simulated deep sequencing data and measured the extent to which the model successfully recovers the true clonal structure of the data.

For each simulation, we began by randomly generating four matrices. First, we generated a simulated matrix 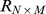 of total read counts with respect to a fixed number (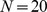) of loci and a fixed number (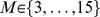) of subsections with a mean coverage of 1000 reads per locus. The matrix was generated by independently sampling each column (corresponding to a single subsection) from a multinomial distribution 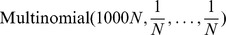, where the parameters 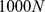 and 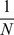 correspond to the total number of trials, and the probability of success for each of the 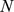 loci, respectively. Second, for any clone number 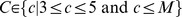, we generated a corresponding Boolean matrix 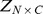, in which the entry at row 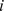 and column 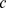 indicates whether locus 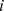 exhibits the variant allele in clone 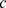. Entries in 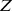 were generated independently from a Bernoulli distribution with a probability of success 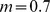, with the exception of the first (“normal”) column of 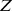, which contains all zeroes. Third, we generated a clone frequency matrix 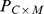 as follows: each element of 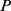 is independently drawn from a Uniform 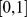 distribution, and then each column of 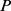 was divided by the column sum, so that the columns summed to 1. We then set 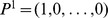 so that the first column of 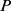 corresponds to the normal subsection. Finally, for each locus 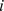 and subsection 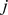, we generated the observed number of variant alleles 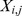 by sampling from a binomial distribution with parameters 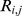 (representing the total number of reads) and 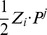 (representing the probability that a given read corresponds to the variant allele). This last step complies with our primary assumption about the distribution of the data (Equation 3).

We ran the EM algorithm using the simulated data 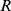 and 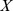 and then evaluated the extent to which the estimated clone frequency matrix 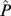 and mutation probability matrix 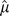 differed from the corresponding true matrices 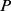 and 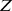. Specifically, we computed the genotype error 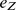, defined as
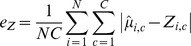
and the clone frequency error 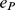,
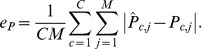



Note that, because we did not know which columns of 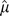 correspond to which columns of 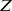, we compared 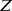 to every permutation of the columns of 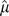 and selected the permutation that resulted in the smallest genotype error. The selected permutation was then also used in the calculation of the clone frequency error.

**Figure 2:**
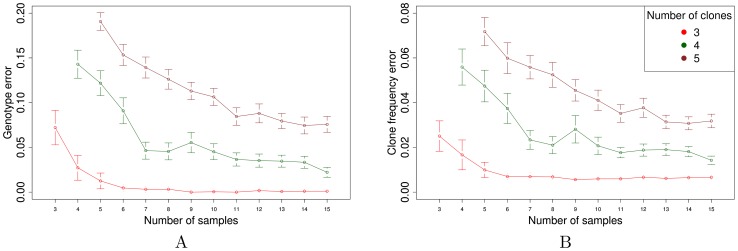
Simulation results. The figure plots the mean (A) genotype error 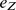 and (B) clone frequency error 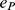 as a function of the number of subsections. Each mean is computed over 100 simulated data sets. For each data set, the EM optimization is repeated from 10 different random initializations, and the results corresponding to the largest log likelihood are reported.

Our simulation results (Figure 2) exhibit two primary trends. The overall error rate, as measured by either genotype or clone frequency error, decreases systematically as the number of subsections increases, and increases as the number of clones increases. Overall, both error rates are low, especially for 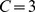. The observed trends are expected: for a fixed number of clones, the availability of more subsections leads to more accurate estimation of the true parameter values; and for a fixed number of subsections, the presence of more clones leads to a greater number of parameters that must be inferred, leading to greater error in estimation.

To assess the affect of sequencing error on the performance of Clomial, we added noise to the simulated data and repeated the above experiments. Specifically, we modeled noise by Bernoulli random variables with probability of success interpreted as the probability that a non-tumor allele is read as a tumor allele or vica versa. Running the EM algorithm on the noisy data revealed that Clomial is robust with respect to noise for all reasonable levels of sequencing error (Figure S6) in Text S1.

### 5 Application to a primary breast cancer

**Figure 3:**
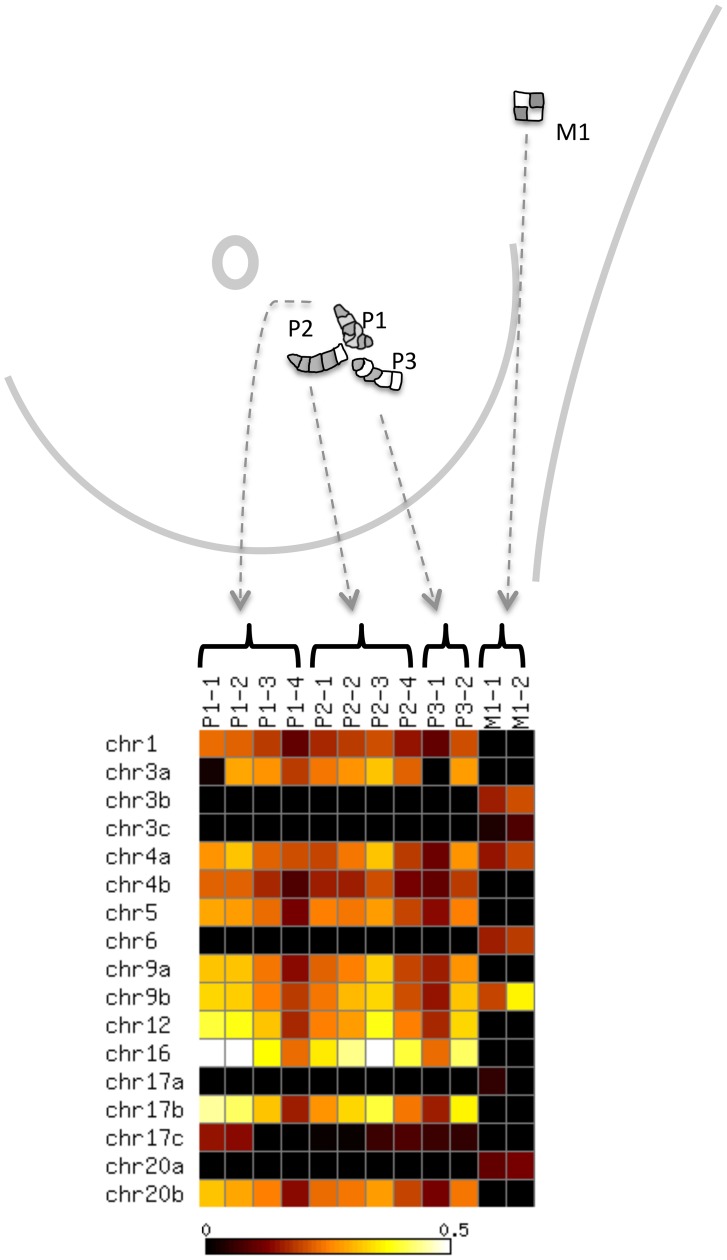
Anatomic locations of the sections, and corresponding allele frequencies. The figure shows (top) the anatomic locations of the three primary and one metastatic sections and (bottom) the corresponding alternative allele frequencies for each subsection. The full coordinates for each of the 17 loci are provided in Dataset S2.

We obtained breast cancer tissue from a 44 year old premenopausal female with infiltrative ductal carcinoma (IDC) with ductal carcinoma in situ (DCIS), stage pT1c pN1, Grade II/III, estrogen receptor (ER) positive, progesterone receptor (PR) positive and Her2 negative. Axillary lymph node dissection revealed that one out of 13 nodes was positive for metastatic disease. A total of 6 tissue sections were obtained, including 2 sections from adjacent normal breast tissue, 3 from the primary breast cancer, and 1 from the positive lymph node. The tumor content, including both IDC and DCIS, ranged from 40% to 55% in the primary tumor and axillary lymph node tissue sections based on pathological examination. For subsequent analysis, each tissue section was subdivided into subsections (Figure 3).

To identify mutations and quantify allele frequencies, we performed two rounds of DNA sequencing. Initially, DNA was extracted from each individual subsection and subjected to exome capture followed by Illumina sequencing. Variants were detected independently in each subsection using the SeattleSeq Annotation Server. We focused on single nucleotide variants and short indels that exhibited a coverage of 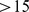 reads in at least one of the subsections, ranking them using DeepSNV [31] and Fisher's exact test (Methods). This analysis produced an initial set of 281 variants (Dataset S1).

To better quantify the allele frequencies at these loci, we designed primer pairs surrounding each locus and used these primers to perform a second round of targeted DNA sequencing. This experiment successfully sequenced 244 of the 281 loci, with a mean and median coverage of 1615 and 1118, respectively, reads per locus. Each of these loci was individually validated by visual inspection using the Integrative Genomics viewer (IGV). Manual inspection showed that many of the initially identified mutations were flanked by homopolymer repeats, suggesting that the alternate alleles were read calling errors, rather than true mutations [32]. For all downstream analysis we focused on a set of 17 confirmed somatic variants. For clarity of presentation, we refer to each somatic variant by the chromosome where it resides, appending a letter if more than one somatic variant occurred within a chromosome (Table S1 in Text S1). The targeted sequencing thus produced two 17-by-12 matrices containing, respectively, the total coverage and the tumor allele count at each locus (Table S1 in Text S1). Visual inspection of the allele frequency profiles shows, not surprisingly, a markedly different pattern of allele frequencies among the subsections from primary and metastatic sites (Figure 3). In addition, several of the samples (e.g., P1-4 and P3-1) exhibit consistently lower frequencies across all loci, presumably indicating a higher prevalence of normal cells within these samples.

**Figure 4:**
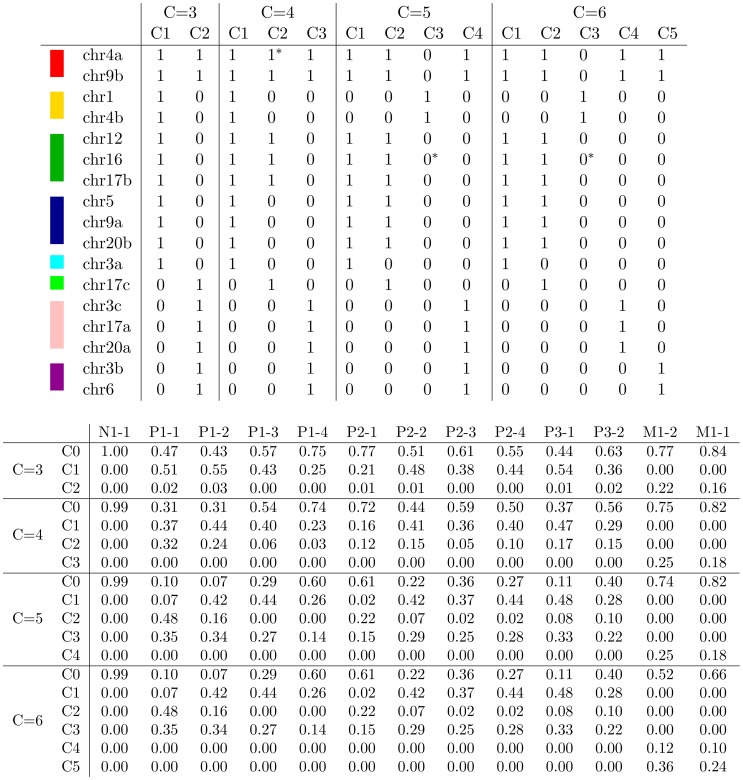
Inferred clonal genotypes and frequencies. The top table lists, for each of the 17 loci, the inferred clonal genotypes using the EM procedure, assuming C = 3, 4, 5 and 6. In each case, the normal clone (C0) is omitted from the inferred matrix 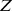, because its genotype consists entirely of zeroes by construction. For reference, each distinct genotype pattern per locus is assigned a unique color according to the scheme from Figure 6. In the table, bits with asterisks were flipped based on the phylogenetic analysis. The corresponding inferred clonal frequencies are listed in the bottom table, where each block shows a matrix 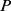 for a value of 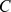, and C0 denotes the normal clone.

We applied our EM optimization procedure to the two counts matrices, varying the number of assumed clones from C = 3 up to C = 6. For each value of C, we ran EM 100,000 times from different random initializations, and we selected the solution with the highest likelihood (Figure 4). The resulting three-clone solution identifies two mutations, chr4a and chr9b, that occur in both the primary and metastatic samples and segregate the remaining mutations into nine that occurred in the primary tumor and six that occurred in the metastatic lymph node. The four- and five-clone solutions further subdivide the primary tumor mutations, and the six-clone solution separates the two metastatic mutations into distinct clones.

**Figure 5:**
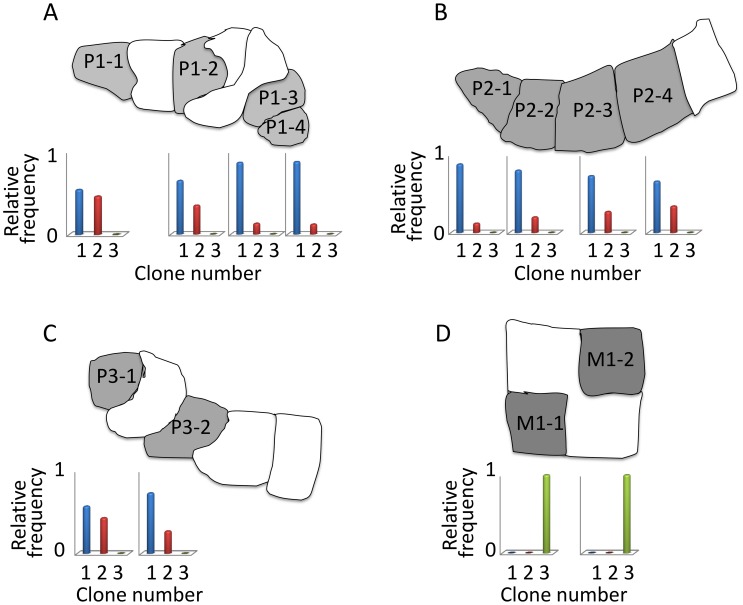
Clone frequencies vary smoothly across adjacent subsections. The panels display the pattern of inferred clone frequencies across subsections (A) P1, (B) P2, (C) P3 and (D) M1. Each bar plot shows the relative frequencies of tumor clones in the corresponding subsection after accounting for normal contamination. Clones are numbered as in Figure 4, and the normal clone, C0, is not shown. This figure shows the 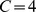 solution; Figure S1 in Text S1 shows the 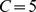 and 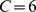 solutions.

To better understand the inferred clonal landscape, we investigated the relationship between clone frequencies and the anatomy of the three primary and one metastatic tumor sections. We hypothesized that clone frequencies should vary smoothly between adjacent subsections, reflecting the physical spread of successful clonal populations. This hypothesis is supported by the data (Figure 5 and Figure S1 in Text S1). The trends are most striking in sections P1 and P2, for which we obtained four separate subsections. In each case, the primary clone frequencies vary in a monotonic fashion as we traverse the sample. Given that the EM inference procedure was provided with no information about which subsection was derived from which section, nor the relative orientation of the subsections to one another, the smoothly varying frequencies among adjacent subsections provides evidence that the method has successfully identified true clonal variation.

### 6 Tumor phylogeny

Cancer progression is an evolutionary process in which clones accrue mutations over time, forming new clones. Accordingly, it should be possible to organize the clonal progression of a tumor into a phylogenetic tree with the founder clone at the root. We therefore investigated whether the clones inferred by our EM procedure obey some simple phylogenetic constraints, with two complementary goals. First, because our EM procedure makes no use of phylogenetic constraints, this analysis can provide further evidence for the validity of our inferred solutions. Second, the phylogenetic analysis has the potential to provide significant insights into the clonal and mutational history of this specific cancer.

**Figure 6:**
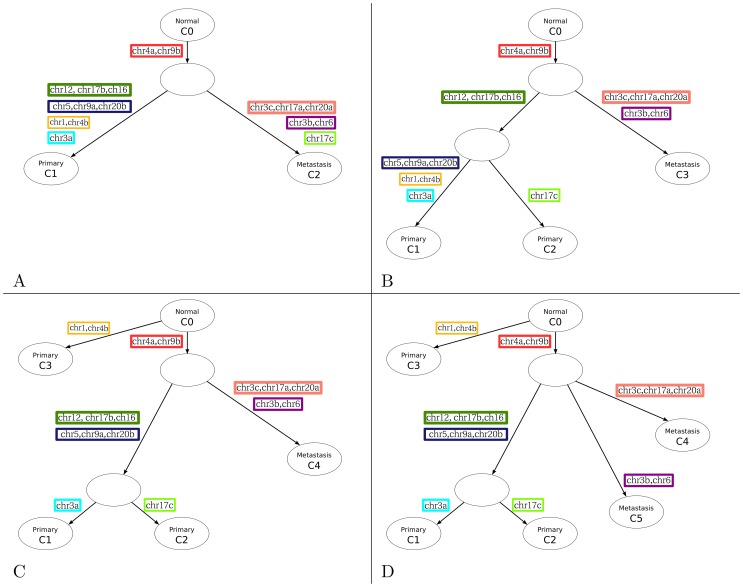
Cancer phylogenies. Each panel shows the inferred clonal phylogeny assuming (A) 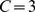, (B) 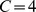, (C) 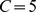 and (D) 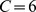 clones, where C0 corresponds to the normal clone. Nodes correspond to inferred clonal populations, and edges are annotated with mutations that occur between the parent and child clones. Two mutations are grouped into a colored box if they both occur on the same branch in all four phylogenies.

We started with the C = 3 solution to our EM algorithm, manually constructing a phylogenetic tree in which each node is a clonal population, and edges are marked with the mutations that occurred in the evolution from the parent clone to the offspring (Figure 6A). This particular tree shows two founder mutations, chr4a and chr9b, occurring prior to metastasis, six mutations occurring along the metastatic lineage, and nine along the primary lineage. This is the only phylogenetic tree that is consistent with the inferred clonal genotypes.

In contrast, for the solutions inferred from the EM algorithm assuming C = 4 through 6, we found that it is not possible to construct a tree without requiring that the same mutation occur independently along multiple branches. We therefore considered all possible “nearby” trees (where “nearby” means that, among the distinct rows of the genotype matrix, the two trees differ by only one bit) that produce a valid phylogenetic tree with no repeated mutations. For example, for the C = 4 solution, we evaluated the likelihood of six nearby trees, yielding log-likelihoods of −28482, −21282, −7500, −6692, −5659, and −4333 (Table S2 in Text S1). The highest of these likelihoods is −4333, compared to −4244 for the solution initially inferred by EM. The selected solution requires changing only one bit in the genotype matrix from “0” to “1” (indicated by asterisks in Figure 4). The resulting phylogenetic tree (Figure 6B) closely resembles the C = 3 tree, except that one mutation initially assigned to the metastatic clone C3 is instead assigned to clone C2 in the C = 4 tree. Also, the nine mutations associated with the primary section in the C = 3 tree are further subdivided into three that occur shortly after metastasis and six that lead to clone C1. Reassuringly, the C = 5 and C = 6 solutions, constructed in a similar fashion (Figure 6C–D), are largely consistent with this story, each introducing a subdivision among the existing sets of mutations to produce a larger set of clones. Among these trees, the only inconsistencies concern (1) three mutations (chr5, chr9a and chr20b) that occur later according to the C = 4 solution than according to the C = 5 or C = 6 solutions and (2) two mutations (chr1 and chr4b) that are assigned their own branch, directly off the normal clone, in the C = 5 and C = 6 solutions. In practice, the chance that a randomly generated genotype matrix would produce a valid phylogenetic tree is vanishingly small (Note S3 in Text S1). Therefore, the fact that each of our inferred solutions very nearly produce a valid phylogenetic tree provides evidence for the validity of these solutions.

We also investigated the extent to which the observed mutation frequencies obey the phylogenetic tree. In principle, a mutation that occurs earlier in the evolution of the cancer should have a higher frequency than mutations that occur later along the same lineage because a child clone necessarily contains all of the mutations belonging to its parent clone. This investigation is hampered, however, by copy number variation. In practice, we cannot directly compare the allele frequencies of two distal sites because the observed allele frequencies are actually the product of mutation frequency and copy number. Empirically, we observe variation in copy number along the genome and differences in copy number variation from one subsection to the next (Figure S2 in Text S1). A consistent duplication of a large portion of chromosome 8 is known to occur commonly in breast cancer [33]. We were lucky, however, that two of our mutated loci occur quite close to one another on chromosome 9 (chr9a and chr9b, separated by only 3.3 Mbp). Given the observed data, the likelihood that a change in copy number occurring between these two loci is small, thereby allowing us to safely compare the corresponding mutation frequencies. Across all nine primary tumor subsections, we observe that the frequency of the parent mutation (chr9b) is higher than that of the child mutation (chr9a). Hence, these mutation frequencies are consistent with the inferred phylogeny.

To assess the stability of our inference, we performed leave-one-out analysis and compared the inferred phylogenies as follows. We held out each of the 12 tumor subsections one at a time and trained the model using the data from only 11 subsections for the case of C = 4. When samples p1-1 or p1-3 were excluded, the inferred genotypes were exactly the same as the genotype obtained from the full data. Excluding any of the other of 10 subsections resulted in a genotype which was different only in one bit; namely, the mutation chr4a was predicted to be present in all clones. However, this difference did not affect the inferred phylogeny because the change of this bit was in fact required to build a valid phylogenetic tree (Figure 4). In other words, by excluding any of the 12 tumor subsections, the inferred genotype always led to the same valid phylogenetic tree, which suggests that our algorithm is stable.

## Discussion

Once a tumor has been resected, clinicians pay a great deal of attention to characterizing its anatomy. Features such as necrosis, extension beyond normal anatomical boundaries, and microvascular invasion convey important prognostic information. In addition, the cancer cells within any given tumor are frequently heterogeneous with respect to features such as differentiation state, the fraction of cells undergoing mitosis (as determined by Ki67 staining), or (for breast cancer) the fraction of cells expressing HER-2 or estrogen receptor. The method described here provides a framework for linking a tumor's molecular anatomy to its structural anatomy as well as its phylogenetic evolution.

Several lines of evidence support the validity of the clonal genotypes and relative frequencies inferred by our model. One prediction from our phylogenetic reconstruction is that somatic variants at the trunk will be present at higher frequencies throughout all tumor subsections than variants appearing at the branches. While copy number variation across the somatic genome complicates these comparisons, one of two closely juxtaposed somatic variants (chr9b) is positioned at the trunk of our phylogenetic tree, while its neighbor (chr9a) arises in one of the branches. Consistent with this representation, the variant allele frequencies for chr9b are consistently higher than for chr9a in all ten tumor subsections examined.

Interestingly, phylogenies can be built from the inferred genotypes even given the relatively low purity of the tumor sections: contamination with normal tissue was 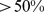 in 9 out of 12 subsections in our data (Figure 4, 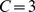). In particular, although we estimate that the metastatic subsections contained 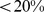 tumor cells in M1-1 and 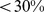 in M1-2, the corresponding branch of the phylogenies is stable and consistent.

Similar to phylogenetic analysis, reassembly of the tumor subsections indicates that our assignment of mutations to clones produces spatial representations that are anatomically reasonable. With further refinements, our method should enable reconstructions that layer a tumor's phylogeny on top of its spatial organization.

While our results underscore the potential power of this new method, our study also has several limitations. Our assessments were confined to heterozygous somatic variants, and did not take into account the many chromosomal structural changes that were present in the tumor we examined. A comparison of exome copy numbers between primary tumor and lymph node indicates that the vast majority of these chromosomal changes preceded the divergence shown in our phylogenetic tree (Figure S2 in Text S1). In theory, one could imagine generalizing our generative model to take copy number variations into account by replacing the 2 in the denominator of Equation 2 with a hidden random variable for each locus, but without some form of aggressive regularization, this formulation would lead to a prohibitively complex and overfit model.

Additionally, a key characteristic of our method is the requirement to specify the number of clones 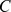 prior to the EM inference procedure. It is important to recognize that this choice should depend upon properties of the data set itself, rather than fundamental properties of the cancer. After all, each cell division results in multiple mutations, such that every cancer cell constitutes a distinct clone. Consequently, a picture of the full clonal history of a cancer would consist of a phylogenetic tree with one leaf for each cancer cell. In practice, such a tree would be of limited utility and, more importantly, could not be accurately estimated from any reasonably sized data set. Perhaps the most useful definition of a tumor clone is a population of cells that exhibit distinct spatial or functional properties. Our approach allows the user to specify the number of clones and, hence, the resolution at which the clonal history is viewed.

Because Clomial does not impose any assumption on the distribution of mutation frequencies, the number of inferred clones may not exceed the number of samples; otherwise, the resulting optimization problem will be under-constrained.

In the particular cancer studied here, the three-clone solution appears to provide an inaccurate view of the clonal history. The placement of the chr17c mutation along the path leading to metastatic clone C2 is surprising, given that this particular locus has such low counts for both metastatic subsections (2 counts for subsection M1-1 and 0 counts for M1-2, Table S1 in Text S1). This apparent anomaly can be explained by the small counts associated with chr17c in four out of the 10 primary tumor subsections (3 counts in P1-3, 4 in P1-4, and 21 in each of P2-1 and P2-2). Faced with the choice of what genotype profile to assign to this particular locus, the inference procedure selected a solution in which only two subsections, rather than four, are inconsistent. However, given the flexibility of a 4-clone model, the anomaly is resolved, and chr17c defines a novel clone C2 that occurs in the primary tumor samples and is completely absent from the metastatic samples.

In practice, it may be possible to estimate how many clones the data set can resolve using a method such as the Bayesian Information Criterion (BIC), with a smaller BIC value indicating a better fit to the data [34]–[36]. This approach has been used previously for estimating tumor clonal composition [37], [38]. BIC analysis of our model on simulated data suggests that, on average, the BIC accurately estimates the true number of clones, even in the presence of sequencing noise (Figure S3A–B in Text S1).

We also computed the BIC for models trained on our real breast cancer data (Figure S3C in Text S1) and observed a large decrease in BIC (45%) when 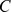 increases from 3 to 4, suggesting that the 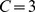 model is too simple to describe the data. However, the subsequent improvements of the BIC are smaller: 29%, 20%, 9%, and 3% respectively, as 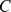 grows from 4 to 8. In general, one should avoid increasing the complexity of the model when the BIC improvement is small because, in such situations, adding to the number of free parameters of the model can potentially lead to over-fitting [39]–[47]. Note that, as an alternative to a BIC approach, one could instead take an approach motivated by cross-validation, as has been explored in the context of matrix factorization models [48]–[50].

Running the EM algorithm is very fast. In practice, using a 2.40 GHz processor with 2 GB memory, training a single EM instance on the real data set takes a few seconds up to several minutes, depending on the value of the hyperparameter 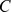 (Figure S4 in Text S1). However, because the optimization problem in the M step is non-convex, many EM instances must be trained from different random initializations to avoid local optima.

We first noted that Clomial achieved good results on simulated data using only 10 random initializations when 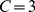 (Figure 2). Then, to further assess the appropriate number of EM instances to run, we revisited the solutions from all of our 100,000 EM instances, counting how many instances are required to achieve the best observed model (Figure S5 in Text S1). In practice, while 1000 EM instances is sufficient to find the optimum solution when 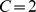 or 3, a larger number of random initializations is required as the number of clones grows. This is an expected phenomenon because the complexity of the model grows significantly with 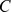, resulting in an optimization surface with many more local optima. Consequently, despite the highly parallel nature of the computation, scaling up to analysis of larger data set with larger numbers of clones will likely require improved EM training strategies, such as noise injection or regularization.

Finally, although we used a simple phylogenetic tree construction procedure to evaluate the quality of our inferred clonal genotypes, the EM inference procedure described here does not explicitly model tumor evolution. Ultimately, we aim to produce a model that automatically infers not only clonal genotypes and clonal frequencies, but also the number of clones and the phylogenetic tree relating them.

Our method differs significantly from other approaches. A recent characterization of 21 breast cancers defined clones by clustering mutations with similar variant allele frequencies [9]. The success of this strategy hinges on characterizing the frequencies of large numbers (hundreds or thousands) of somatic variants. In contrast, our method can reconstruct clonal phylogenies based on accurately measuring alleles of much smaller numbers of somatic variants. The view afforded by our method may provide novel insights into tumor biology. In particular, results from Nik-Zainal and colleagues [9] were interpreted to indicate that cancers become clinically apparent only after one of the competing clones has achieved clonal dominance. In contrast to this “winner takes all” hypothesis, our model suggests that some cancers might be more accurately regarded as ecosystems, in which clones may be subject to spatial influences that affect their competitive fitness, or may even collaborate to support tumor growth.

An important difference between our method and many other methods based on clustering [8], [9], [12] is our explicit probabilistic modeling of the random selection of normal and variant alleles during sequencing, according to a binomial distribution. By taking into account not just the relative frequency of the two alleles but the separate counts of normal and variant alleles, our model automatically assigns less importance to a locus with lower coverage, even if the locus yields the same variant allele frequency as a high-coverage locus.

While this manuscript was under review, two methods called PyClone [13] and PhyloSub [51] were published, which do model allele counts using a binomial distribution. These methods attempt to simultaneously infer not only clonal genotypes and frequencies, as Clomial does, but also infer the number of clones and their phylogeny. Furthermore, PyClone and PhyloSub are not limited, as Clomial is, to situations in which the number of inferred clones is less than or equal to the number of available samples. How is this possible? To make these inferences feasible, these clustering methods must make certain distributional assumptions about the data. Specifically, PyClone assumes a Dirichlet Process prior for clone frequencies, where the base distribution is Uniform 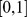 and the concentration parameter is Gamma distributed with shape and scale parameters equal to 1 and 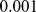, respectively. PhyloSub extends PyClone by using a tree-structured stick-breaking process [52] to directly account for phylogenetic relationships during the inference. In principle, these assumptions enable PyClone and PhyloSub to infer information about a large number of clones from only a single sample. On the other hand, when multiple samples are available, Clomial can draw accurate inferences without requiring these distributional assumptions. In practice, our comparison showed that Clomial and PhyloSub produce similar results on three previously described chronic lymphocytic leukemia (CLL) cases [53] (Tables S3–S5 in Text S1).

We note that if 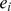 is the sequencing error rate at locus 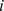, then the probability of observing a variant allele at this locus in subsection 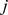 is estimated by 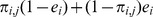. In principle, sequencing noise could be incorporated into our model by replacing 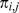, defined in Equation 2, with 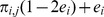 in the likelihood and EM algorithm. However, given the robustness of the current method to noise (Figures S6 and S3C in Text S1), we opted to keep our model simple. In future applications, it may be beneficial to model noise in data produced by sequencing technologies that exhibit high error rates (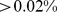) such as PacBio RS [54].

The EM algorithm is not the only option for maximizing the log-likelihood for the observed data. In particular, one could instead treat both 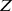 and 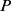 as optimization variables and seek to maximize 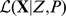 with respect to 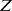 and 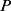. This would amount to iteratively updating 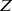 and then updating 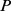 until convergence, similar to the iterative algorithms typically used for matrix factorization models [23]–[25], [50]. However, this alternative approach would not have any computational advantage in terms of the update for 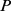, which would still not have a closed-form solution, and would need to be solved using BFGS-B or an equivalent approach. Furthermore, the update for 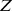 would be very complicated under the constraint that 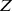 is a binary matrix. Therefore, we developed a customized inference algorithm based on EM.

Whereas genetic testing for cancer patients today focuses on mutations affecting a relatively small number of cancer-associated genes, most cancers are sustained by networks of aberrantly regulated genes that collaborate to promote tumor growth. The ability to assign mutations to clones, and to layer a tumor's clonal content on top of its structural anatomy in space and over time, can provide new insights into the mechanisms that enable cancers to invade, metastasize and escape treatment.

## Materials and Methods

### Ethics statement

This research was reviewed and approved by the Cancer Consortium Institutional Review Board (IRB) located at the Fred Hutchinson Cancer Research Center (FHCRC). The FHCRC has an approved Federalwide Assurance on file with the Office for Human Research Protections (number 00001920). The Federalwide Assurance is a formal written, binding commitment that assures that the FHCRC promises to comply with the regulations and ethical guidelines governing research with human subjects, as stipulated by the U.S. Department of Health and Human Services under 45 CFR 46. Because this study involved the use of de-identified specimens obtained from an IRB-approved repository, we did not interface with patients. Patient consent was administered, in compliance with 45 CFR 46, by investigators who maintain the repository. Patients gave their consent for their specimens to be stored in the repository and subsequently used for research in cancer. The FHCRC IRB deemed that our research was in concordance with the purpose of the registry and the patient informed consent.

### Breast cancer tissue sample

We obtained breast cancer tissues from the Breast Cancer Biospecimen Repository of Fred Hutchinson Cancer Research Center after IRB approval. The patient was a 44 year old pre-menopausal woman diagnosed with infiltrative ductal carcinoma (IDC) and ductal carcinoma in situ (DCIS), stage pT1c pN1, Grade II/III, ER positive, PR positive and Her2 negative. Axillary lymph node dissection revealed that one out of 13 nodes was positive for metastatic disease. A total of 5 pieces were obtained from surgical samples including 1 tissue section from adjacent normal breast tissue (N1), 3 tissue sections from the primary breast cancer (P1, P2, P3), and 1 tissue section from the positive axillary lymph node (M1). Each section is about 1 cm by 1 cm by 0.5 cm. The tumor content, including both IDC and DCIS, ranges from 40% to 55% in the primary tumor and axillary lymph node tissue sections based on pathological examination (P1 55% IDC, P2 45% IDC, P3 40% IDC and 15% DCIS, M1 50% IDC).

### Tissue DNA extraction

Each individual section was subdivided into multiple subsections, and the anatomic locations of all the subsections were recorded (Figure 3). Using Qiagen AllPrep DNA/RNA Micro Kit, DNA was extracted from one normal subsection (N1-1), seven primary subsections (P1-2, P1-3, P1-5, P2-1, P2-3, P3-3, P3-4) and one metastatic subsection (M1-1). After quantification, all the DNA samples were subjected to exome capture followed by Illumina sequencing.

### Whole exome sequencing

Next generation sequencing was carried out at the Northwest Genome Center at University of Washington on the normal subsection, seven primary subsections, and one metastatic subsection. For each subsection, one microgram of genomic DNA was used to construct the random-shearing library per standard protocol with Covaris acoustic sonication. Libraries then underwent exome capture using the 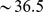 Mb target from Roche/Nimblegen SeqCap EZ v2.0 (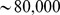 exons and flanking sequence). Since each library was uniquely barcoded, samples were performed in multiplex. Massively parallel sequencing was carried out on the HiSeq sequencer.

### Read processing

Sequence reads were processed with a pipeline consisting of the following elements: (1) base calls generated in real-time on the HiSeq instrument (RTA 1.12.4.2); (2) Perl scripts developed in-house to produce demultiplexed fastq files by lane and index sequence; (3) demultiplexed BAM files aligned to a human reference (hg19) using BWA (Burrows-Wheeler Aligner; v0.5.9) [55]. Read-pairs not mapping within 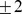 standard deviations of the average library size (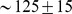 bp for exomes) are removed. All aligned read data were subjected to the following steps: (1) “duplicate removal” was performed, (i.e., the removal of reads with duplicate start positions; Picard MarkDuplicates; v1.14); (2) indel realignment was performed (GATK IndelRealigner; v1.0-6125) resulting in improved base placement and lower false variant calls; (3) base qualities were recalibrated (GATK TableRecalibration; v1.0-6125). All sequence data then underwent a previously described quality control protocol [56].

### Variant detection

Variant detection and genotyping were performed using the UnifiedGenotyper tool from GATK (v1.0-6125). Variant data for each sample were formatted (variant call format) as “raw” calls that contain individual genotype data for one or multiple samples, and flagged using the filtration walker (GATK) to mark sites that are of lower quality/false positives, e.g., low quality scores (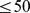), allelic imbalance (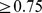), long homopolymer runs (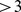) and/or low quality by depth (QD 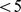).

### Calling single nucleotide variants (SNVs) and indels

Most of the commonly used software for calling SNVs and indels, including SNVMix [57] and VarScan [58], requires tumor content 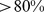. To allow identification of low frequency alleles that occur in only one or a few subsections, we did not pool all of the data together. Instead, we designed a method that is appropriate for multiple samples from one patient, with relatively low tumor content, ranging from 45% to 55%. At each chromosomal position (locus), we considered six mutually exclusive possible outcomes: A, C, G, T, deletion, and unknown. The counts of these six outcomes at each locus between normal and each of the multiple tumor subsections were compared with a 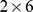 Fisher's exact test. To correct for multiple testing, we used the **qvalue** R package to convert 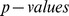 to 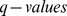. Only those chromosomal loci with 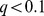 in at least one comparison between normal and tumor samples were accepted for downstream analysis. This analysis identified 6310 loci.

For each accepted locus, we used a heuristic procedure to identify which of the six alleles differed between the tumor and normal sample. For each subsection, we carried out six 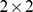 Fisher's exact tests, one for each of the six possible alleles. Thus, each such test compared one allele's counts to the sum of the counts for the other five alleles. Using a p-value threshold of 0.01, an allele was declared to be increased, decreased, or unchanged in the tumor subsection as compared to the normal sample. The changes that were classified as “increased” and had a normal count of zero were called tumor-specific mutations. This procedure identified a total of 268 such tumor-specific mutations, with a mean and median sequencing depth of 92 and 75, respectively. Corresponding annotations were obtained from SeattleSeq (http://snp.gs.washington.edu/SeattleSeqAnnotation137).

In parallel, we also analyzed our data using deepSNV [31] by comparing the normal subsection to the 8 tumor subsections. We ran deepSNV on the loci with total coverage across all samples more than 50, which resulted in the identification of 29 loci with 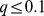. The union of the two lists yielded 281 loci for further validation (Dataset S1).

### Targeted deep sequencing

Mutations were validated by targeted deep sequencing of DNA derived from one normal subsection (N1-1), 10 primary subsections (P1-1, P1-2, P1-3, P1-4, P2-1, P2-2, P2-3, P2-4, P3-1, P3-2) and two metastatic subsections (M1-1 and M1-2). The subsections were selected to have low normal content and to span the tumor anatomy. Genomic DNA was prepared as described for the initial exome sequencing. A HaloPlex probe capture library for selective capture of 281 target loci was generated with SureDesign (Agilent Technologies). Target enrichment for deep sequencing was carried out with the HaloPlexTM Target Enrichment System from Agilent Technologies following the manufacturer's protocol. Triplicate enrichments were performed for each sample. Target-enriched samples were sequenced using a MiSeq (Illumina). Of the 281 target loci, 244 were successfully sequenced with coverage more than 100 reads for the normal sample. The mean, median, and the standard deviation of the coverage were 1615, 1118, and 1600, respectively (Dataset S2).

All 244 loci were visualized using the Integrative Genomics Viewer [59], [60]. A set of 17 loci were selected based upon three criteria: (1) at least 3 reads cover the locus in the normal sample, (2) the variant allele is not present in the normal tissue (allowing for a few variant counts, which may reflect sequencing error) and (3) there are no nearby clustered mutations, indicative of sequencing or mapping error. Independently, the data were also analyzed using deepSNV. Applying a 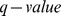 threshold of 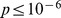 yielded 19 loci, including all 17 of the initially selected loci. The 17 loci were retained for downstream analysis (Table S1 in Text S1).

### Bayesian Information Criterion analysis

We computed BIC using the following formula:
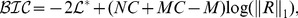
(14)where 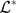 is the expectation of the complete-data log likelihood, which is maximized in the last M step (see Equations 7 and 13). Also, 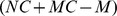 represents the total number of free parameters, and 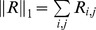 is the total number of counts.

## Supporting Information

Dataset S1Dataset S1 includes, for each of the 281 targeted loci, the following information: (1) the chromosomal coordinates of the locus, (2) the variant allele, (3) for the 268 targeted loci identified by Fisher's exact test, the number of reads supporting the mutation and the coverage of the locus in each subsection, (4) the 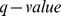 from the 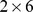 Fisher's exact test, (5) the minimum 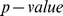 from the six 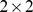 Fisher's exact tests, and (6) the q-value for each of the 29 target regions identified by deepSNV.(XLS)

Dataset S2Dataset S2 lists, for each of the 281 sequenced target regions, the following information: (1) the chromosomal coordinates of the locus, (2) the integer counts for each of the five possible alleles (A, C, G, T, -, where “-” denotes deletion or insertion) in each of the ten primary subsections, two metastatic subsections, and the normal subsection, (3) for each subsection, the deepSNV 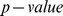 for the test that the subsection has a mutation on each specific locus, and (4) the mnemonic for each of the 17 mutations used in our inference procedure.(XLS)

Software S1 Software S1 is an R package called *Clomality* that implements the EM algorithm described in this paper.
(GZ) Click here for additional data file.

Text S1 Text S1 includes five supplementary notes, six supplementary figures and five supplementary tables.
(PDF)
