# Supplementary material for: Inferring Clonal Composition from Multiple Sections of a Breast Cancer
Source: PLoS Comput Biol. 2014 Jul 10;10(7):e1003703. doi: 10.1371/journal.pcbi.1003703 (PMC4091710; doi:10.1371/journal.pcbi.1003703)
Supplement: Software S1 — is an R package called Clomality that implements the EM algorithm described in this paper. (GZ) [file pcbi.1003703.s003.gz › Clomial/inst/doc/Clonal_decomposition_by_Clomial.pdf]

Clomial: A likelihood maximization approach to infer the clonal structure of a cancer using multiple tumor samples

Habil Zare

February 28, 2014

Contents

1 Introduction **2**

1.1 Oncological motivation 2

1.2 Computational methodology 2

2 How to run Clomial? **2**

2.1 An example 3

2.2 Choosing the best model 4

3 Reference **5**

1 Introduction

Identifying the clonal decomposition of a tumor is oncologically important and computationally challenging.

1.1 Oncological motivation

Cancers arise from successive rounds of mutation and selection, generating clonal populations that vary in size, mutational content and drug responsiveness. Ascertaining the clonal composition of a tumor is therefore important both for prognosis and therapy. Mutation counts and frequencies resulting from next-generation sequencing (NGS) potentially reflect a tumor's clonal composition; however, deconvolving NGS data to infer a tumor's clonal structure presents a major challenge.

1.2 Computational methodology

Clomial is based on a generative model for NGS data derived from multiple subsections of a single tumor. The counts of alternative alleles are modeled using binomial distributions, and therefore, the novel method is called **"Clomial"**; **Clo**nal decomposition using bino**mial** models. Unlike the previous approaches, Clomial is capable of incorporating multiple samples in the analysis together to increase statistical power. It uses an expectation-maximization (EM) procedure for estimating the clonal genotypes and relative frequencies as described in *Zare et al. 2014*, which demonstrates, via simulation, the validity of Clomial approach. As a real life example, this package also provides the data and code to infer the clonal composition of a primary breast cancer and associated metastatic lymph node. Applying Clomial to larger numbers of tumors should cast light on the clonal evolution of cancers in space and time.

Please refer to *Zare et al. 2014* for a complete description of the methodology, and the results on the real data set.

2 How to run Clomial?

Clomial is an R package source that can be downloaded from BioCunductor. In Linux, it can be installed by the following command:

```
R CMD INSTALL Clomial_x.y.z.tar.gz
```

where x.y.z. determines the version.

The main function is `Clomial()` which takes as input 2 matrices Dt and Dc. They contain the counts of the alternative allele, and the total number of processed reads, accordingly. Their rows correspond to the genomic loci, and their columns correspond to the samples. Also, C, the assumed number of clones should be provided to `Clomial()`. Several models should be trained using different initial values to escape from local optima. Then, the best one in terms of the likelihood can be chosen by choose.best() function. Finally, the Bayesian Information Criterion (BIC) can be performed using the compute.bic() function to estimate the number of clones. However, the results of BIC analysis should be interpreted with great caution for the reasons explained in *Zare et al. 2014*.

2.1 An example

This example shows how Clomial can be run on counts obtained from next generation sequencing. The user might use standard tools such as BWA, Samtools, or DeepSNV to map the reads and compute the counts. In the following example, we analyze ready-to-use data from a primary breast cancer and associated metastatic lymph node, where breastCancer is a list containing the input 2 matrices Dt and Dc.

```
> library(Clomial)
> set.seed(1)
> data(breastCancer)
> Dc <- breastCancer$Dc
> Dt <- breastCancer$Dt
> Clomial2 <-Clomial(Dc=Dc,Dt=Dt,maxIt=20,C=4,binomTryNum=2)
```

Clomial is done and the results are stored in `Clomial2`. We are interested in `Clomial[["models"]]` which is a list of trained models. For each trained model, Mu models the matrix of genotypes, where rows and columns correspond to genomic loci and clones, accordingly. Also, `P` is the matrix of clonal frequency where rows and columns correspond to clones and samples, accordingly. For example, the trained parameters for the first model are given by:

```
> model1 <- Clomial2$models[[1]]
> ## The clonal frequencies:
> round(model1$P,digits=2)

   A5-2 A1-1 A1-4 A1-3 A1-2 A2-1 A2-3 A3-1 A3-2 A3-3 A3-4 B1-1 B1-2
C1 0.99 0.12 0.08 0.38 0.63 0.60 0.28 0.42 0.27 0.15 0.31 0.82 0.87
C2 0.00 0.40 0.39 0.21 0.14 0.19 0.26 0.21 0.32 0.32 0.35 0.00 0.00
C3 0.00 0.03 0.03 0.00 0.00 0.02 0.01 0.00 0.00 0.02 0.02 0.18 0.13
C4 0.00 0.45 0.50 0.41 0.23 0.19 0.45 0.36 0.41 0.51 0.32 0.00 0.00

> ## Similarly, the genotypes is given by round(model1$Mu).
```

2.2 Choosing the best model

In practice, more models should be trained using multiple random initialization to escape from local optima. This can be done by, say, `binomTryNum=1000` which needs longer computational time, in order of two hours, for this data. For the purpose of this manual, we can use pre-computed results as follows:

```
> data(Clomial1000)
> models <- Clomial1000$models
> ## Number of trained models:
> length(models)

[1] 1000
```

The best model in terms of likelihood is given by `choos.best()` function:

```
> chosen <- choose.best(models=models, doTalk=TRUE)

[1] "Choosing the best out of 1000 ..."
[1] 1000

> M1 <- chosen$bestModel
> print("Genotypes according to the best model:")

[1] "Genotypes according to the best model:"

> round(M1$Mu)
```

```
                C1 C2 C3 C4
chr12:111884756  0  0  1  1
chr16:7743333    0  0  1  1
chr9:139352016   0  1  1  1
chr17:10404056   0  0  1  1
chr20:33764653   0  0  0  1
chr9:136081779   0  0  0  1
chr4:187250053   0  0  0  1
chr3:132219738   0  0  0  1
chr1:117620562   0  0  0  1
chr4:39606599    0  1  0  1
chr5:140215480   0  0  0  1
chr6:51907756    0  1  0  0
chr17:34913028   0  0  1  0
chr20:2639398    0  1  0  0
chr3:190165603   0  1  0  0
chr3:193201749   0  1  0  0
chr17:10369879   0  1  0  0
```

```
> print("Clone frequencies in the first sample:")
```

```
[1] "Clone frequencies in the first sample:"
```

```
> round(M1$P[,1],digits=2)
```

```
  C1   C2   C3   C4
0.99 0.00 0.00 0.00
```

In the above demo example, the number of clones was assumed to be 4. For real applications, it is useful to try several clone numbers and compare the results using BIC. Also, note that fitting the best set of parameters needs a large number of models to be trained on data in order to escape from local optima. In practice, the suitable number of models depends on the assumed number of clones, number of genomic loci, and number on samples.

3 Reference

Zare et al., **Inferring clonal composition from multiple sections of a breast cancer**, Under review.
