# Supplementary material for: Inferring Clonal Composition from Multiple Sections of a Breast Cancer
Source: PLoS Comput Biol. 2014 Jul 10;10(7):e1003703. doi: 10.1371/journal.pcbi.1003703 (PMC4091710; doi:10.1371/journal.pcbi.1003703)
Supplement: Text S1 — includes five supplementary notes, six supplementary figures and five supplementary tables. (PDF) [file pcbi.1003703.s004.pdf]

Supplement to "Inferring clonal composition from multiple sections of a breast cancer"

Habil Zare[1,*], Junfeng Wang[2,*], Alex Hu[1], Kris Weber[1], Josh Smith[1], Debbie Nickerson[1], ChaoZhong Song[2], Daniela Witten[3,†], C. Anthony Blau[2,†], and William Stafford Noble[1,4,†]

[1]Department of Genome Sciences, University of Washington, Seattle, WA
[2]Division of Hematology, Department of Medicine, University of Washington, Seattle, WA
[3]Department of Biostatistics, University of Washington, Seattle, WA
[4]Department of Computer Science and Engineering, University of Washington, Seattle, WA

Note S1

Lemma 1.1 *Suppose $f : \mathbb{R}^n \to \mathbb{R}$ is a real valued function with n parameters $x_1, \ldots, x_n$. Consider the optimization problems*

$$\begin{cases} \hat{x} = \underset{x}{\operatorname{argmax}}\, f(x_1, \ldots, x_n) \\ \text{subject to } 0 \leq x_i, \displaystyle\sum_i x_i < 1 \end{cases} \tag{S1}$$

and

$$\begin{cases} \hat{v} = \underset{v}{\operatorname{argmax}}\, f\left(\dfrac{v_1}{1+\displaystyle\sum_i v_i}, \ldots, \dfrac{v_n}{1+\displaystyle\sum_i v_i}\right) \\ \text{subject to } 0 \leq v_i \end{cases}. \tag{S2}$$

There is a one-to-one mapping between their solutions:

$$\hat{x_i} = \frac{\hat{v_i}}{1+\displaystyle\sum_i \hat{v_i}}, 1 \leq i \leq n. \tag{S3}$$

Proof First, notice that the mapping (S3) is one-to-one, since

$$\hat{v_i} = \frac{\hat{x_i}}{1-\sum \hat{x_i}}, 1 \leq i \leq n.$$

Second, notice that $\frac{\hat{v_i}}{1+\sum_i \hat{v_i}}$ is in the feasible set of (S1), since $\frac{\hat{v_i}}{1+\sum_i \hat{v_i}} > 0$ and $\sum_{i=1}^{n} \frac{\hat{v_i}}{1+\sum_i \hat{v_i}} < 1$ for $\hat{v_i} \geq 0$. The result follows directly. ∎

Note S2

Note that from Equation 2 we have:

$$\frac{\partial \pi_{i,j}}{\partial P_{c,j}} = \frac{\partial(\frac{1}{2}\mathbf{Z}_i \cdot P^j)}{\partial P_{c,j}} = \frac{1}{2}\mathbf{Z}_{i,c}.$$

In what follows, $\Phi : \mathbb{R}^{C\times M} \to \mathbb{R}$ is a multivariable function of P defined in Equation 9. Also, for any fixed $1 \le j \le m$, $P_{c,j} : \mathbb{R}^{C-1} \to \mathbb{R}$ is a function of $(V_{2,j}, \ldots, V_{C,j})$ as defined in Equation S15. Because $P_{c,j'}$ does not depend on $V_{c,j}$ if $j' \neq j$, the partial derivative $\frac{\partial\Phi}{\partial V_{c,j}}$ depends only on $P_{c',j}$ for $1 < c' \le C$. We have:

$$
\begin{aligned}
\frac{\partial \Phi}{\partial V_{c,j}} &= \sum_{c'=2}^{C}\left(\frac{\partial \Phi}{\partial P_{c',j}} \cdot \frac{\partial P_{c',j}}{\partial V_{c,j}}\right) \\
&= \sum_{c'=2}^{C}\left(\left(\sum_{i=1}^{N}\frac{\partial \Phi}{\partial \pi_{i,j}} \cdot \frac{\partial \pi_{i,j}}{\partial P_{c',j}}\right) \cdot \frac{\partial P_{c',j}}{\partial V_{c,j}}\right) \\
&= \frac{1}{2}\sum_{c'}\left(\left(\sum_{i,z}\left(q_{\mathbf{Z},i}\left(\frac{\mathbf{X}_{i,j}}{\pi_{i,j}} - \frac{R_{i,j}-\mathbf{X}_{i,j}}{1-\pi_{i,j}}\right)\mathbf{Z}_{i,c'}\right)\right) \cdot \frac{\partial}{\partial V_{c,j}}\left(\frac{V_{c',j}}{1+\sum_{1<c''} V_{c'',j}}\right)\right) \\
&= \sum_{c'}\left(\left(\sum_{i,z}\left(q_{\mathbf{Z},i}\mathbf{Z}_{i,c'}\left(\frac{\mathbf{X}_{i,j}}{\pi_{i,j}} - \frac{R_{i,j}-\mathbf{X}_{i,j}}{1-\pi_{i,j}}\right)\right)\right) \cdot \frac{(1+\sum_{1<c''} V_{c'',j})\mathbb{1}_c(c') - V_{c',j}}{2(1+\sum_{1<c''} V_{c'',j})^2}\right)
\end{aligned}
$$

where $\pi_{i,j} = \frac{1}{2}\mathbf{Z}_i \cdot P^j$, and the identity function $\mathbb{1}_c(c') = 1$ if $c' = c$, and it is zero otherwise.

Note S3

To estimate the probability of obtaining a random genotype matrix that corresponds to a valid phylogenetic tree, we randomly generated one million such matrices, independently setting each bit to 1 or 0 by a fair coin flip. We then asked whether, for each matrix, it is possible to generate a corresponding phylogenetic tree. This is the case if, for each pair of rows in the genotype matrix, the bitwise "AND" of the rows either (1) consists of all zeroes, or (2) is equal to one of the rows. Out of one million 17×3 matrices, 2.2% were phylogenetically consistent. The corresponding percentages for 17×4 and 17×5 matrices were 0.0097% and 0.0001%.

Note S4

The complete-data log likelihood, which was defined in Equation 4 in the main text, can be computed as follows. Because loci are independent of each other, we can write $\mathcal{L}$ as the sum of the log likelihood associated with each locus. Then

$$
\begin{aligned}
\mathcal{L} &= \log \Pr(\mathbf{X}, \mathbf{Z}|\theta) \\
&= \sum_{1\le i\le N} \log \Pr(\mathbf{X}_i, \mathbf{Z}_i|\theta) \\
&= \sum_{1\le i\le N} \log\Big(\Pr(\mathbf{X}_i|\mathbf{Z}_i,\theta)\Pr(\mathbf{Z}_i|\theta)\Big) \qquad \text{(S4)} \\
&= \sum_{1\le i\le N} \log\Big(\Pr(\mathbf{X}_i|\mathbf{Z}_i,\mu_i,P)\Pr(\mathbf{Z}_i|\mu_i,P)\Big) \qquad \text{(S5)} \\
&= \sum_{1\le i\le N}\bigg(\log\Big(\Pr(\mathbf{X}_i|\mathbf{Z}_i,\mu_i,P)\Big) + \log\Big(\Pr(\mathbf{Z}_i|\mu_i,P)\Big)\bigg). \qquad \text{(S6)}
\end{aligned}
$$

Equations S4 and S5 are obtained by conditional probability and the definition of θ, respectively. Note that conditioned on μ_i, the random variable $\mathbf{Z}_i$ is independent from P; therefore, $\Pr(\mathbf{Z}_i|\mu_i, P) = \Pr(\mathbf{Z}_i|\mu_i)$. Similarly, $\Pr(\mathbf{X}_i|\mathbf{Z}_i, \mu_i, P) = \Pr(\mathbf{X}_i|\mathbf{Z}_i, P)$. Then

$$\mathcal{L} = \underbrace{\sum_i \log \Pr(\mathbf{X}_i|\mathbf{Z}_i, P)}_{\mathcal{L}_X} + \underbrace{\sum_i \log \Pr(\mathbf{Z}_i|\mu_i)}_{\mathcal{L}_Z}. \tag{S7}$$

We compute each of the two terms in Equation S7 separately, starting with the second term, $\mathcal{L}_Z$, which is simpler to compute. Recalling that each entry $\mathbf{Z}_{i,c}$ is an independent Bernoulli random variable with parameter $\mu_{i,c}$, we have

$$\begin{aligned}
\mathcal{L}_Z &= \sum_{1\leq i\leq N} \log \Pr(\mathbf{Z}_i|\mu_i) \\
&= \sum_{1\leq i\leq N} \log \left(\prod_{1\leq c\leq C} \Pr(\mathbf{Z}_{i,c}|\mu_{i,c}) \right) \\
&= \sum_{i,c} \log \left((\mu_{i,c})^{\mathbf{Z}_{i,c}} (1-\mu_{i,c})^{1-\mathbf{Z}_{i,c}} \right) && \text{(S8)} \\
&= \sum_{i,c} \left(\mathbf{Z}_{i,c} \log(\mu_{i,c}) + (1-\mathbf{Z}_{i,c}) \log(1-\mu_{i,c}) \right). && \text{(S9)}
\end{aligned}$$

To compute $\mathcal{L}_X$, we use the assumption of independence between subsections to get

$$\begin{aligned}
\mathcal{L}_X &= \sum_i \log \Pr(\mathbf{X}_i|\mathbf{Z}_i, P) \\
&= \sum_{1\leq i\leq N} \log \left(\prod_{1\leq j\leq M} \Pr(\mathbf{X}_{i,j}|\mathbf{Z}_i, P^j) \right) \\
&= \sum_{i,j} \log \Pr(\mathbf{X}_{i,j}|\mathbf{Z}_i, P^j).
\end{aligned}$$

Note that conditioned on $\mathbf{Z}$ and P, for any locus i and subsection j, $\mathbf{X}_{i,j}$ is a binomial random variable with parameters $R_{i,j}$ and $\pi_{i,j} = \frac{1}{2}\mathbf{Z}_i \cdot P^j$ (Equations 2 and 3). Also, the total number of reads, $R_{i,j}$, is known from the experiment. Therefore, we have

$$\begin{aligned}
\mathcal{L}_X &= \sum_{i,j} \log \Pr(\mathbf{X}_{i,j}|\mathbf{Z}_i, P^j) \\
&= \sum_{i,j} \left(\log \binom{R_{i,j}}{\mathbf{X}_{i,j}} + \mathbf{X}_{i,j} \log(\pi_{i,j}) + (R_{i,j} - \mathbf{X}_{i,j}) \log(1-\pi_{i,j}) \right). && \text{(S10)}
\end{aligned}$$

Finally, substituting Equations S9 and S10 into Equation S7 yields the following formula for the complete-data log likelihood:

$$\begin{aligned}
\mathcal{L} &= \mathcal{L}_X + \mathcal{L}_Z \\
&= \sum_{i,j} \left(\log \binom{R_{i,j}}{\mathbf{X}_{i,j}} + \mathbf{X}_{i,j} \log(\pi_{i,j}) + (R_{i,j} - \mathbf{X}_{i,j}) \log(1-\pi_{i,j}) \right) \\
&\quad + \sum_{i,c} \left(\mathbf{Z}_{i,c} \log(\mu_{i,c}) + (1-\mathbf{Z}_{i,c}) \log(1-\mu_{i,c}) \right),
\end{aligned}$$

where $\pi_{i,j} = \frac{1}{2}\mathbf{Z}_i \cdot P^j$.

Note S5

P^{new} is the solution of the following constrained optimization problem:

$$\begin{cases} P^{\text{new}} := \underset{P}{\operatorname{argmax}}\, \Phi(P) \\ \text{such that } \forall j, c : 0 \le P_{c,j} \text{ and } \forall j : \sum_{c=1}^{C} P_{c,j} = 1. \end{cases} \tag{S11}$$

To simplify $\Phi(P)$, we let $q_{\mathbf{Z},i}$ be the the posterior probability for locus i, defined as

$$q_{\mathbf{Z},i} := \Pr(\mathbf{Z}_i|\mathbf{X}_i, \theta^{\text{old}}). \tag{S12}$$

By substituting binomial distributions from Equation 8, we get:

$$\begin{aligned} \Phi(P) &= \mathbb{E}_{\mathbf{Z}|\mathbf{X},\theta^{\text{old}}}\left[\log \Pr(\mathbf{X}|\mathbf{Z}, P)\right] \\ &= \mathbb{E}_{\mathbf{Z}|\mathbf{X},\theta^{\text{old}}}\left[\sum_i \log \Pr(\mathbf{X}_i|\mathbf{Z}_i, P)\right] \\ &= \sum_{i=1}^{N} \mathbb{E}_{\mathbf{Z}_i|\mathbf{X}_i,\theta^{\text{old}}}\left[\log \Pr(\mathbf{X}_i|\mathbf{Z}_i, P)\right] \\ &= \sum_{i=1}^{N} \sum_{z\in\{0,1\}^C} \left(q_{\mathbf{Z},i} \log \Pr(\mathbf{X}_i|\mathbf{Z}_i = z, P)\right) \\ &= \sum_{\substack{i=1,j=1 \\ z\in\{0,1\}^C}}^{i=N,j=M} \left(q_{\mathbf{Z},i}\left(\log \binom{R_{i,j}}{\mathbf{X}_{i,j}} + \mathbf{X}_{i,j}\log(\pi_{i,j}) + (R_{i,j} - \mathbf{X}_{i,j})\log(1-\pi_{i,j})\right)\right) \end{aligned} \tag{S13}$$

where $\pi_{i,j} = \frac{1}{2}\mathbf{Z}_i \cdot P^j$.

We point out several facts that help in maximizing $\Phi(P)$:

- All terms other than π in Equation S13 are fixed. In particular, $q_{\mathbf{Z},i}$ is known because it is a function of θ^{old}.
- Because Equation S13 is a summation over the values of j from 1 to M, and the samples are assumed to be independent, maximization can be done by solving M independent problems.
- Because the first column of $\mathbf{Z}$ corresponds to normal contamination, $\mathbf{Z}_{i,1} = 0$ for the ith locus. This means that $\pi_{i,j}$ and therefore Φ are constant with respect to P^1, the first column of P. However, since we have assumed that each column of P sums to 1, there is a simple relationship between $P_{1,j}$ and $P_{2,j}, \ldots, P_{C,j}$:

$$P_{1,j} = 1 - \sum_{1<c} P_{c,j}.$$

- Recall that we assumed $P_{1,j} > 0$ due to contamination with normal cells in each subsection. So for $1 \le j \le M$, the optimization problem (S11) can be written in this form:

$$\begin{cases} P^{j\,\text{new}} := \underset{P^j}{\operatorname{argmax}} \sum_{i=1}^{N} \sum_{z\in\{0,1\}^C} \left(q_{\mathbf{Z},i}\left(\log\left(\binom{R_{i,j}}{\mathbf{X}_{i,j}}\right) + \mathbf{X}_{i,j}\log(\frac{1}{2}\mathbf{Z}_i \cdot P^j) + (R_{i,j} - \mathbf{X}_{i,j})\log(1 - \frac{1}{2}\mathbf{Z}_i \cdot P^j)\right)\right) \\ \text{such that } \forall j, 1 < c : 0 \le P_{c,j} \text{ and } \forall j : \sum_{1<c} P_{c,j} < 1. \end{cases} \tag{S14}$$

We use Lemma 1.1 in Note S1 to solve Equation S14 by a change of variables,

$$P_{c,j} := \frac{V_{c,j}}{1 + \sum_{1<c' \leq C} V_{c',j}}, 1 \leq j \leq M, 1 < c \leq C, \tag{S15}$$

which eliminates the need for the constraint $\sum_{1<c} P_{c,j} < 1$ provided that $V_{c,j} \geq 0$. For simplicity of notation, we set $V_{1,j} = 0$ so that $V_{C \times M}$ has the same dimension as $P_{C \times M}$. The choice of 0 is arbitrary because $V_{1,j}$ has no role in Equation S15.

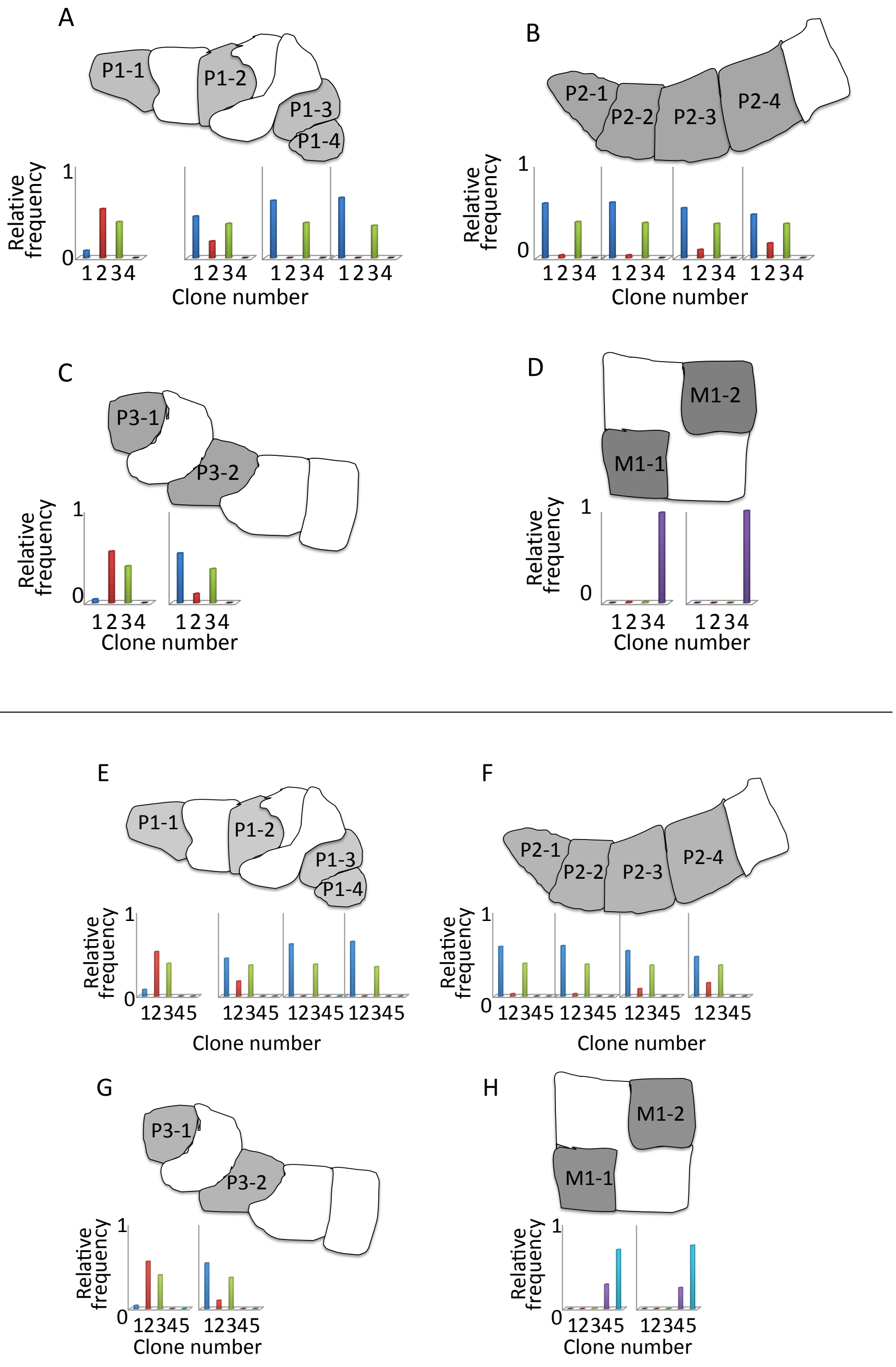

Figure S 1: **Clone frequencies vary smoothly across adjacent subsections.** Each panel displays, for a different section, the pattern of inferred clone frequencies across subsections. Each bar plot shows the relative frequencies of tumor clones in the corresponding subsection after accounting for normal contamination. Clones are numbered as in Figure 4, and the normal clone, C0, is not shown. Panels A–D show the C=5 solution, and panels E–H show the C=6 solution.

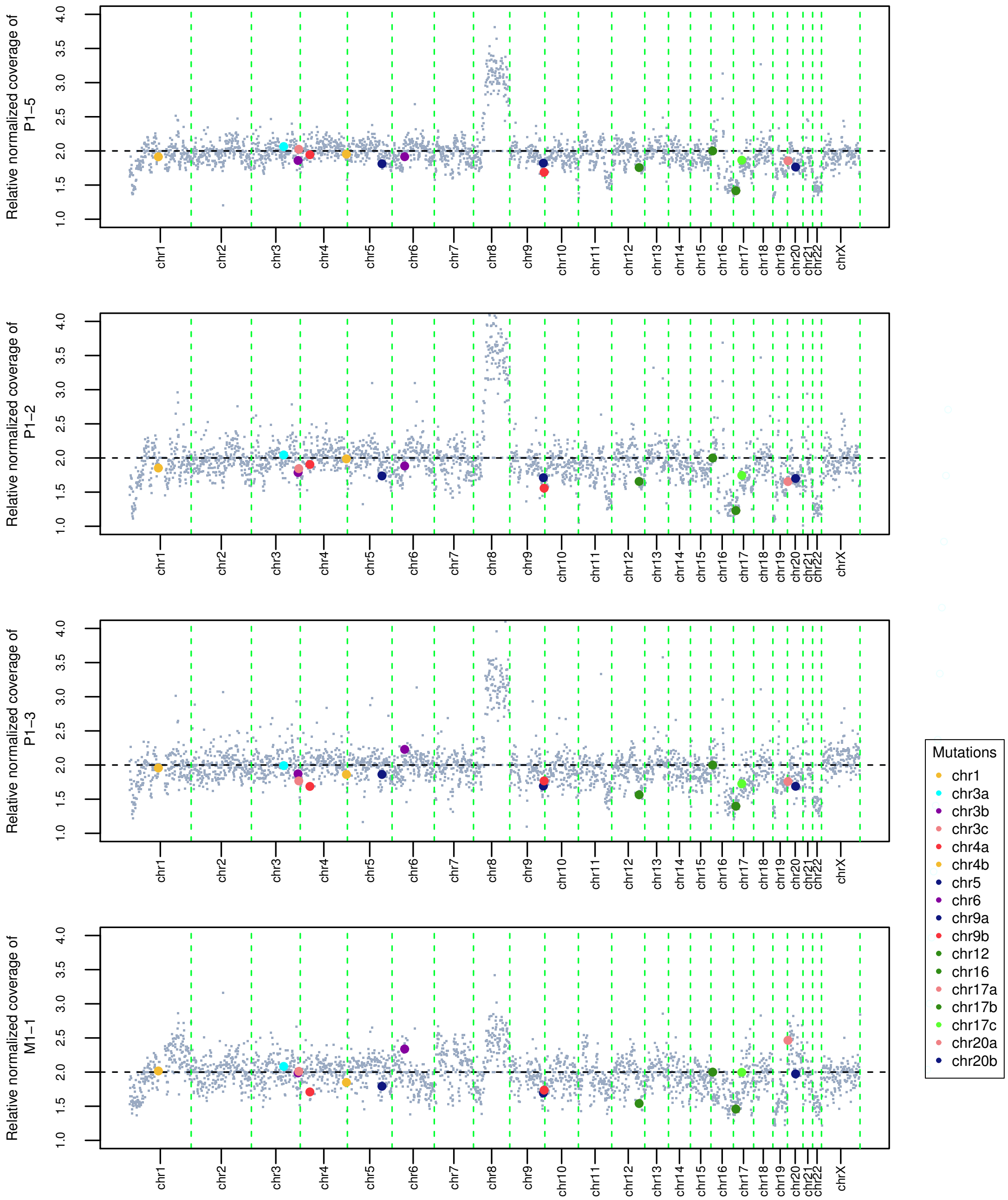

Figure S 2: **Copy number variation across samples.** Each panel shows, for a specified primary tumor subsection, the relative normalized coverage observed per 1 Mbp window (i.e., the ratio of counts in the window over the total counts from a tumor subsection, divided by the corresponding ratio for the normal sample). The 17 loci analyzed in this study are indicated with colored dots. Note that the dot for locus chr17a is occluded by the dot for the adjacent locus chr17b. The color scheme here is the same as in Figure 6 The 17 loci analyzed in this study are indicated with colored dots using the color scheme from Figure 6. Note that the dot for locus chr17a is occluded by the dot for the adjacent locus chr17b.

(A)

(B)

(C)

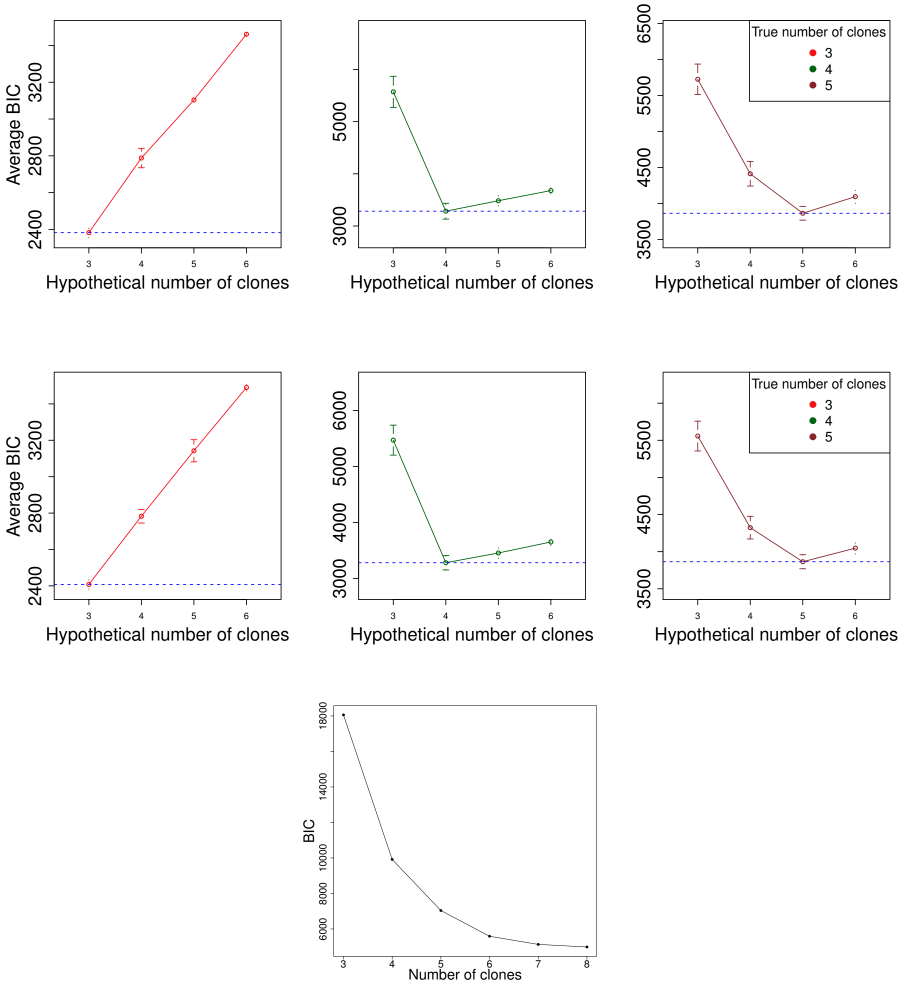

Figure S 3: BIC analysis. (A) Each panel plots the average BIC over 100 simulated data sets (y-axis) as a function of the hyperparameter C, the assumed number of clones (x-axis). Similar to Figure 2, the true number of clones for each panel is shown by colors in the legend. In each case, the minimum average BIC is achieved at the true clone number. (B) Adding sequencing noise with rate 1% to the simulated data does not affect the performance of BIC. (C) BIC values are shown for models trained on our real breast cancer data. While the BIC exhibits a large decrease (45%) when C increases from 3 to 4, the subsequent improvements of the BIC are smaller: 29%, 20%, 9%, and 3%, respectively, as C grows from 4 to 8.

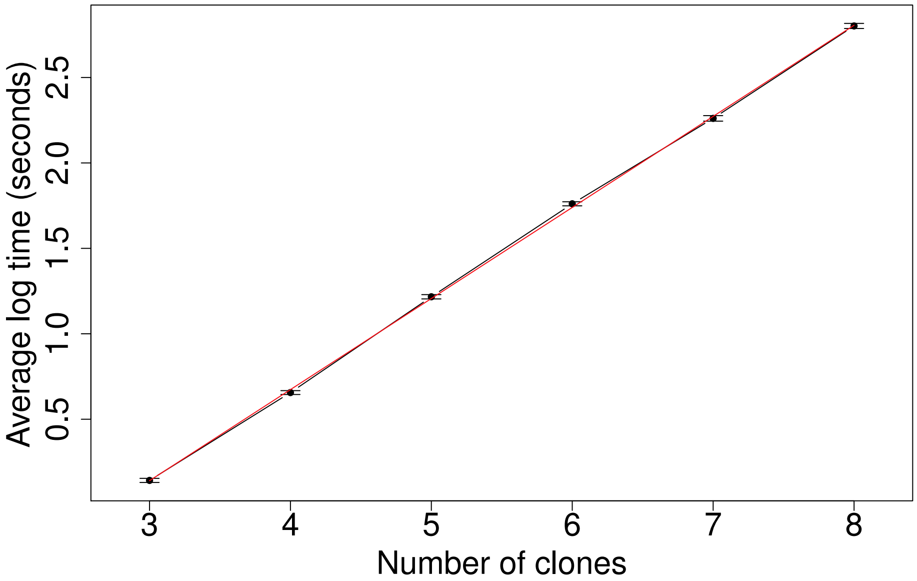

Figure S 4: **Computational time.** The average time for EM training to converge on the breast cancer data (y-axis) is plotted as a function of the hyperparameter C, the assumed number of clones (x-axis). The y-axis is on a $\log_{10}$ scale, and the red line is a linear fit which shows that the training time grows exponentially with C. Each training task was obtained using a 2.40GHz processor with 2 GB memory. Values are averaged over 100 EM runs, trained from different random initializations.

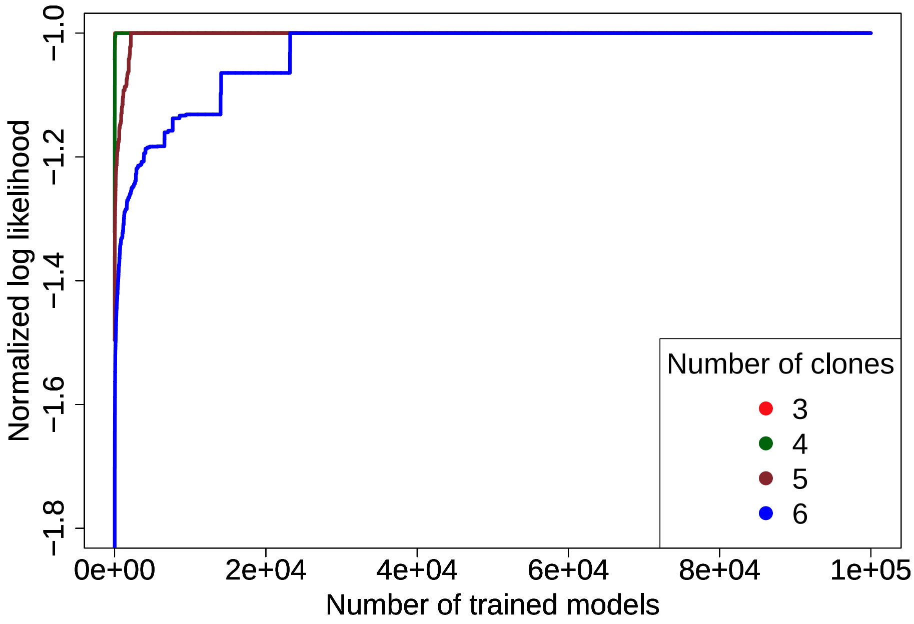

Figure S 5: **Improving likelihood by multiple initializations.** The figure plots, for different values of C, the best log likelihood obtained as a function of the number of EM runs. Specifically, for any k number of EM instances (x-axis), the normalized log-likelihood (y-axis) was obtained by dividing the best log-likelihood of k models by absolute value of the maximum observed log-likelihood. The reported value is the median over 1000 random orderings of a fixed set of 100,000 likelihoods. The curve corresponding to 3 clones is covered by the 4-clones curve because they both reach their optimum likelihood using a relatively small (< 1000) number of initializations.

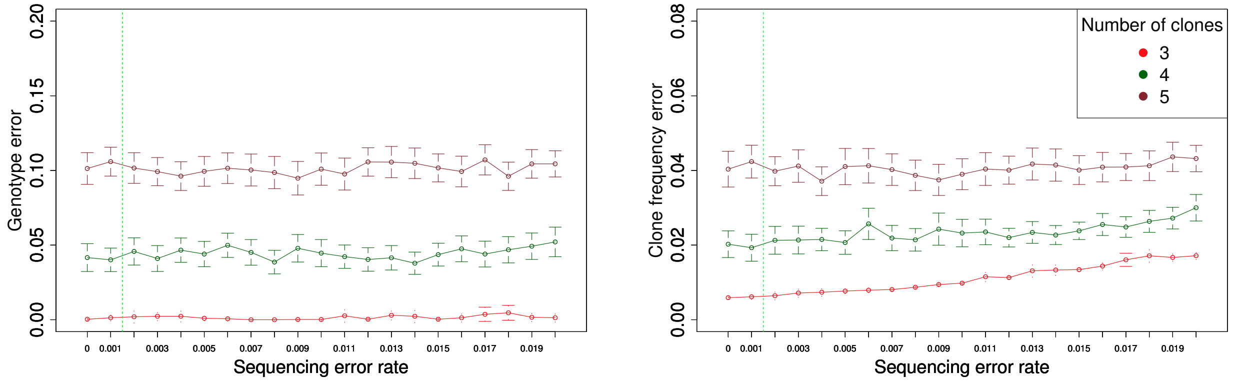

(A) (B)

Figure S 6: **The effect of sequencing noise on the performance of Clomial.** The figure plots the mean (A) genotype error e_Z and (B) clone frequency error e_P as a function of sequencing error rate. The experiments were run as described in Figure 2 using 10 simulated subsections, and the sequencing error rate is the probability that a non-tumor allele is read as tumor allele or vica versa. (A) The genotype error is not affected by sequencing noise less than 0.02. (B) The change in frequency error is negligible when the noise is less than 0.01. The green vertical lines correspond to a sequencing error rate of 0.0015, which is the estimated empirical error rate from our deep sequencing experiment, estimated by averaging the frequency of the tumor alleles in the normal subsections (Figure 4).

	Primary tumor										Metastasis		Normal
	P1-1	P1-2	P1-3	P1-4	P2-1	P2-2	P2-3	P2-4	P3-1	P3-2	M1-1	M1-2	N1-1
chr1	416	519	358	202	286	340	490	256	188	366	1	0	4
chr3a	73	969	696	517	546	621	903	471	13	596	0	0	1
chr3b	0	0	1	1	0	0	0	0	0	0	228	230	0
chr3c	0	0	1	0	2	2	0	0	2	0	122	179	2
chr4a	144	294	163	148	173	229	341	173	96	197	112	169	6
chr4b	665	962	619	321	508	531	863	391	310	541	6	2	3
chr5	251	328	209	118	175	194	329	181	100	203	1	0	2
chr6	0	0	0	0	0	0	0	0	0	0	461	479	0
chr9a	571	755	419	265	485	518	851	483	337	495	2	0	6
chr9b	604	958	500	364	583	718	1101	566	338	691	541	1120	3
chr12	1246	2024	1213	618	1196	1252	2297	1109	752	1438	2	3	27
chr16	1341	1883	1007	596	820	1086	1591	1018	495	1046	6	1	11
chr17a	0	2	0	1	0	0	0	0	0	0	217	0	1
chr17b	713	900	592	358	440	612	841	421	314	621	2	0	7
chr17c	172	240	3	1	21	21	128	134	114	87	2	0	2
chr20a	0	0	0	0	0	0	2	0	0	0	322	401	3
chr20b	609	933	624	304	628	627	967	550	320	563	2	2	2
chr1	2144	2898	2382	2627	2108	2281	2844	2193	2290	2165	3107	2447	2791
chr3a	2981	4085	3217	3475	2747	2868	3479	2654	2901	2540	3981	2900	3080
chr3b	1659	2005	1478	1883	1280	1520	1559	1279	1511	1199	1772	1384	1390
chr3c	2813	3535	3016	3439	2865	3071	3534	2764	2865	2598	3645	2740	3255
chr4a	646	1120	894	870	1073	1166	1293	1157	1040	911	930	1075	1304
chr4b	3745	5438	4420	4591	4009	4021	4980	4042	4109	3688	5438	4592	4802
chr5	1035	1396	1100	1157	844	965	1407	1105	944	965	1438	1301	1233
chr6	2498	3056	2515	2713	2201	2494	3154	2557	2517	2445	3595	3302	2627
chr9a	2197	2874	2131	2358	2643	2477	3194	3033	2686	2220	2670	2750	2826
chr9b	2121	3591	2356	2445	2912	2828	3953	3293	2753	2697	3490	3791	3194
chr12	3758	6235	4701	4520	5834	5332	7200	5409	5343	5201	5435	5413	6007
chr16	3141	4245	3248	3188	2844	2979	3826	3044	2606	2978	3406	2729	3070
chr17a	3136	4169	3774	4474	3430	3408	4431	3726	3880	3113	5066	3941	5200
chr17b	1934	2560	2320	2794	2034	2150	2513	2134	2500	2077	2855	2387	2831
chr17c	1432	2271	2209	2061	2247	2204	2705	2013	2130	1975	2930	2617	2670
chr20a	2295	3479	2536	2680	2703	2628	3611	3035	2739	2623	4010	4039	3318
chr20b	2371	3828	2943	2684	3367	3175	4151	3374	3154	2856	4126	4413	3876

Table S 1: **Allele counts.** The table lists the number of observed tumor allele reads (top) and total number of reads (bottom) per locus, for all 17 loci. These two matrices constitute the entire input to the EM estimation procedure.

	Inferred			M_1			M_2			M_3			M_4			M_5			M_6		
	C1	C2	C3	C1	C2	C3	C1	C2	C3	C1	C2	C3	C1	C2	C3	C1	C2	C3	C1	C2	C3
**chr4a**	**1**	**0**	**1**	0*	0	1	1	1*	1	1	0	0*	1	0	1	1	0	1	1	0	1
chr9b	1	1	1	1	1	1	1	1	1	1	1	1	1	1	1	1	1	1	1	1	1
chr1	1	0	0	1	0	0	1	0	0	1	0	0	1	0	0	1	0	0	1	0	0
chr4b	1	0	0	1	0	0	1	0	0	1	0	0	1	0	0	1	0	0	1	0	0
**chr12**	**1**	**1**	**0**	1	1	0	1	1	0	1	1	0	0*	1	0	1	0*	0	1	1	1*
**chr16**	**1**	**1**	**0**	1	1	0	1	1	0	1	1	0	0*	1	0	1	0*	0	1	1	1*
**chr17b**	**1**	**1**	**0**	1	1	0	1	1	0	1	1	0	0*	1	0	1	0*	0	1	1	1*
chr5	1	0	0	1	0	0	1	0	0	1	0	0	1	0	0	1	0	0	1	0	0
chr9a	1	0	0	1	0	0	1	0	0	1	0	0	1	0	0	1	0	0	1	0	0
chr20b	1	0	0	1	0	0	1	0	0	1	0	0	1	0	0	1	0	0	1	0	0
chr3a	1	0	0	1	0	0	1	0	0	1	0	0	1	0	0	1	0	0	1	0	0
chr17c	0	1	0	0	1	0	0	1	0	0	1	0	0	1	0	0	1	0	0	1	0
chr3c	0	0	1	0	0	1	0	0	1	0	0	1	0	0	1	0	0	1	0	0	1
chr17a	0	0	1	0	0	1	0	0	1	0	0	1	0	0	1	0	0	1	0	0	1
chr20a	0	0	1	0	0	1	0	0	1	0	0	1	0	0	1	0	0	1	0	0	1
chr3b	0	0	1	0	0	1	0	0	1	0	0	1	0	0	1	0	0	1	0	0	1
chr6	0	0	1	0	0	1	0	0	1	0	0	1	0	0	1	0	0	1	0	0	1
log-likelihood	-4,244			-21,282			**-4,333**			-5,659			-28,482			-7,500			-6,692		

Table S 2: Modified genotypes and the corresponding likelihoods. Genotype for C=4 after any of the above modifications is done. The inferred genotype, and all of the six possible modifications are shown, where each asterisk indicates a flipped bit. The associated log-likelihoods are reported. The phylogeny tree presented in Figure 6C was made based on modification M_2 which had the highest likelihood.

	Clomial				Manual				PhyloSub			
	C1	C2*	C3	C4	C1	C2	C3	C4	C1	C2	C3	C4
BCL2L13	1	0*	1	1	1	1	1	1	1	1	1	1
COL24A1	0	1	0	0	0	1	0	0	0	1	0	0
DAZAP1	0	0	1	1	0	0	1	1	0	0	1	1
EXOC6B	0	0	1	1	0	0	1	1	0	0	1	1
GHDC	0	0	1	1	0	0	1	1	0	0	1	1
GPR158	1	0*	1	1	1	1	1	1	1	1	1	1
HMCN1	0	1	0	0	0	1	0	0	0	1	0	0
KLHDC2	0	1	0	0	0	1	0	0	0	1	0	0
LRRC16A	0	0	0	1	0	0	0	1	0	0	0	1
MAP2K1	0	1	0	0	0	1	0	0	0	1	0	0
NAMPTL	1	0*	1	1	1	1	1	1	1	1	1	1
NOD1	0	1	0	0	0	1	0	0	0	1	0	0
OCA2	0	0	1	1	0	0	1	1	0	0	1	1
PLA2G16	0	0	1	1	0	0	1	1	0	0	1	1
SAMHD1	1	1	1	1	1	1	1	1	1	1	1	1
SLC12A1	1	0*	1	1	1	1	1	1	1	1	1	1

		a	b	c	d	e
	C0	0.00	0.00	0.00	0.00	**0.00**
	C1	0.27	0.18	0.16	0.23	**0.43**
Clomial	C2*	0.01	0.03	0.04	0.12	0.34
	C3	0.39	0.27	0.30	0.26	0.13
	C4	0.33	0.52	0.50	0.39	0.10
	C0	0.08	0.03	0.00	0.04	0.38
	C1	0.20	0.15	0.16	0.17	0.08
Manual	C2	0.00	0.03	0.04	0.14	0.31
	C3	0.39	0.27	0.30	0.26	0.13
	C4	0.33	0.52	0.50	0.39	0.10
	C0	0.09	0.03	0.01	0.04	0.40
	C1	0.15	0.17	0.17	0.17	0.04
PhyloSub	C2	0.00	0.03	0.03	0.13	0.32
	C3	0.39	0.19	0.33	0.28	0.16
	C4	0.37	0.58	0.46	0.38	0.08

Table S 3: **Comparing different methods on the CLL077 data set.** The top table lists, for each of the 16 loci of CLL077, the clonal genotypes inferred by Clomial, manual analysis carried out by Shuh et al. [1], and PhyloSub. In each case, the normal clone (C0) is omitted because its genotype consists entirely of zeroes. Any bit predicted not the same as the manual analysis is marked by an asterisk in the genotypes matrices. The corresponding inferred clonal frequencies are listed in the bottom table, where each block shows a matrix P derived by one of the three methods and C0 denotes the normal clone. Frequencies that differ by more than 0.1 from the manual estimates are in bold. All three methods predict the same genotypes for clones C1, C3, and C4. Unlike manual analysis and PhyloSub, Clomial does not consider C2* to be a subclone of C1. Instead, it identifies C2* by a set of mutations which are present mostly in sample e, the most recent one (data not shown). The frequencies from all the three methods are generally close.

	Clomial				Manual				PhyloSub			
	C1	C2	C3*	C4*	C1	C2	C3	C4	C1	C2	C3	C4
ADAD1	1	1	1	1	1	1	1	1	1	1	1	1
AMTN	0	1	0	1*	0	1	0	0	0	1	0	0
APBB2	0	1	0	1*	0	1	0	0	0	1	0	0
ASXL1	1	0	0	1*	1	0	0	1	1	0	0	1
ATM	0	1	1*	1*	0	1	0	0	0	1	0	0
BPIL2	0	1	0	1*	0	1	0	0	0	1	0	0
CHRNB2	1	0	0	0	1	0	0	0	1	0	0	0
CHTF8	1	1	1	1	1	1	1	1	1	1	1	1
FAT3	1	0	0	0	1	0	0	0	1	0	0	0
HERC2	1	1	1	1	1	1	1	1	1	1	1	1
IL11RA	1	1	0*	1	1	1	1	1	1	1	1	1
MTUS1	0	1	0	1*	0	1	0	0	0	1	0	0
MUSK	1	0	0	1	1	0	0	1	1	0	0	1
NPY	1	0	0	0	1	0	0	0	1	0	0	0
NRG3	1	0	0	0	1	0	0	0	1	0	0	0
PLEKHG5	0	1	0	1*	0	1	0	0	0	1	0	0
SEMA3E	1	0	0	1*	1	0	0	1	1	0	0	1
SF3B1	1	1	1	1	1	1	1	1	1	1	1	1
SHROOM1	1	1	1	1	1	1	1	1	1	1	1	1
SPTAN1	0	1	0	1*	0	1	0	0	0	1	0	0

		a	b	c	d	e
	C0	0.00	0.00	0.35	0.09	0.03
	C1	0.00	0.01	0.46	0.91	0.96
Clomial	C2	0.72	0.84	**0.01**	0.00	0.00
	C3*	**0.18**	0.08	0.06	0.00	0.00
	C4*	0.10	0.07	0.12	0.01	0.01
	C0	0.06	0.02	0.30	0.06	0.02
	C1	0.00	0.01	0.44	0.89	0.96
Manual	C2	0.82	0.91	0.13	0.00	0.00
	C3	0.03	0.00	0.00	0.00	0.00
	C4	0.09	0.06	0.13	0.05	0.03
	C0	0.08	0.00	0.36	0.08	0.01
	C1	0.00	0.00	0.43	0.92	0.99
PhyloSub	C2	0.79	0.86	0.11	0.00	0.00
	C3	0.00	0.07	0.00	0.00	0.01
	C4	0.13	0.07	0.10	0.00	**-0.01**

Table S 4: **Comparing different methods on the CLL003 data set.** The tables lists, for each of the 20 loci of CLL003, the clonal genotypes inferred by Clomial, manual analysis, and PhyloSub, using notation similar to Table S3. All three methods agree on the genotypes of the dominant clones C1 and C2, which have relatively high frequencies. Also, the corresponding frequencies for these clones, and also the normal clone, are similar between the three inference methods. However, Clomial does not agree with the manual analysis on the genotypes of C3* and C4*. PhyloSub incorrectly predicts a negative frequency for clone C4 in sample e.

	Clomial				Manual					PhyloSub					
	C1	C2	C3	C4*	C1	C2	C3	C4	C5	C1	C2	C3*	C4	C5	C6*
ARHGAP29	1	1	1	1	1	1	1	1	1	1	1	1	1	1	1
EGFR	1	1	1	1	1	1	1	1	1	1	1	1	1	1	0
IRF4	0*	0	0	1	1	0	0	0	1	1	0	0	0	1	0
KIAA0182	1	1	1	1	1	1	1	1	1	1	1	1	1	1	1
KIAA0319L	1	0	0	1	1	0	0	1	1	1	0	0	1	1	0
KLHL4	1	1	1	1	1	1	1	1	1	1	1	1	1	1	1
MED12	1	1	1	1	1	1	1	1	1	1	1	1	1	1	1
PILRB	1	1	1	1	1	1	1	1	1	1	1	1	1	1	1
RBPJ	1	0	0	0	1	0	0	0	0	1	0	0	0	0	0
SIK1	1	1	1	1	1	1	1	1	1	1	1	1	1	1	1
U2AF1	1	1	0	1	1	1	0	1	1	1	1	0	1	1	0

		a	b	c	d	e
Clomial	C0	0.00	0.00	0.00	0.00	0.00
	C1	0.03	0.09	0.03	0.13	0.12
	C2	0.36	0.31	0.36	0.22	0.13
	C3	0.32	0.09	0.30	0.06	0.09
	C4*	0.30	0.52	0.31	0.58	0.66
Manual	C0	0.00	0.00	0.00	0.00	0.00
	C1	0.02	0.09	0.03	0.13	0.13
	C2	0.31	0.31	0.32	0.22	0.17
	C3	0.32	0.09	0.30	0.06	0.09
	C4	0.11	0.08	0.11	0.15	0.04
	C5	0.24	0.43	0.24	0.44	0.57
PhyloSub	C0	0.00	0.00	0.00	0.00	0.00
	C1	0.02	0.08	0.03	0.14	0.13
	C2	0.33	0.37	0.36	0.22	0.16
	C3*	0.23	0.05	0.20	0.04	0.05
	C4	0.13	0.03	0.08	0.17	0.05
	C5	0.23	0.45	0.26	0.41	0.56
	C6*	0.06	0.02	0.07	0.02	0.05

Table S 5: **Comparing different methods on the CLL006 data set.** The tables lists, for each of the 11 loci of CLL006, the clonal genotypes inferred by Clomial, manual analysis, and PhyloSub, using notation similar to Table S3. All three methods predict the same genotypes for clones C1, C2, and C3, except mutation of IRF4 which is absent in C1 according to Clomial. The corresponding frequencies are also very close, if we assume that PhyloSub splits C3 from the manual analysis to C3* and C6*. Given only five samples, Clomial can infer a maximum of five clones, including the normal clone. Therefore, Clomial merges C4 and C5 from the manual analysis to C4*, as is evident from comparison of the corresponding genotypes and frequencies. All three methods agree that the normal contamination in all samples is less than 1%.

References

[1] A. Schuh, J. Becq, S. Humphray, A. Alexa, A. Burns, R. Clifford, S. M. Feller, R. Grocock, S. Henderson, I. Khrebtukova, et al. Monitoring chronic lymphocytic leukemia progression by whole genome sequencing reveals heterogeneous clonal evolution patterns. *Blood*, 120(20):4191–4196, 2012.
